# The transmembrane domain of Frey1 harbors a transplantable inhibitory motif for intramembrane proteases

**DOI:** 10.1007/s00018-023-04823-7

**Published:** 2023-06-01

**Authors:** Whendy Contreras, J. Fernando Bazan, Torben Mentrup

**Affiliations:** 1 Institute of Physiological Chemistry, Technische Universität Dresden, Fiedlerstraße 42, 01307 Dresden, Germany; 2 Unit for Structural Biology, VIB-UGent Center for Inflammation Research, Ghent, Belgium

**Keywords:** Intramembrane proteolysis, Signal peptide peptidase-like proteases, γ-Secretase, Enzyme inhibitors, Alzheimer’s disease, Protease regulation

## Abstract

Although aspartic intramembrane-cleaving proteases (I-CLIPs) are crucial switches of multiple signaling pathways and involved in several devastating diseases, little is known about their physiological regulation. We have recently identified Frey regulator of sperm-oocyte fusion 1 (Frey1) as an inhibitory protein of Signal Peptide Peptidase-like 2c (SPPL2c), a member of this protease family. Employing structure modeling along with cell-based inhibition and interaction studies, we identify a short motif within the Frey1 transmembrane domain essential for inhibition of SPPL2c. Intriguingly, this motif can be transplanted to the SPPL2c substrate PLN, thereby transforming it into an inhibitor of this enzyme. It can be adopted for the generation of Notch1-based γ-Secretase inhibitors demonstrating its versatile use among aspartic I-CLIPs. In summary, we describe a mechanism of aspartic I-CLIP inhibition which allows the targeted generation of specific inhibitors of these enzymes and might enable the identification of endogenous negative regulators of these enzymes.

**Supplementary Information:**

The online version contains supplementary material available at 10.1007/s00018-023-04823-7.

## Introduction

Intramembrane-cleaving proteases (I-CLIPs) are specialized enzymes that can cleave peptide bonds in the hydrophobic environment of lipid bilayers [1]. They can be found in all taxa and within eukaryotic cells in most subcellular compartments demonstrating the high conservation and relevance of these enzyme for living organisms [2]. Despite the very different conditions for proteolysis in aqueous or membranous environment, these proteases employ the same catalytic mechanisms as their soluble counterparts and can be grouped into the same enzyme families. Beyond serine proteases (rhomboids), metalloproteases (Site 2 protease) and glutamyl proteases (Rce1), the family of aspartyl I-CLIPs is perhaps the best characterized subgroup of intramembrane proteases [3]. In addition to presenilin (PS) 1 and PS2, that represent the catalytically active subunits of the heterotetrameric γ-Secretase complex [4], this family is composed of Signal Peptide Peptidase (SPP) as well as the four SPP-like (SPPL) proteases SPPL2a, SPPL2b, SPPL2c and SPPL3 [5, 6]. While for many of these enzymes we have just started to discern their physiological functions, aspartic proteases have been identified as central switches in several (patho-)physiological pathways. For example, by processing Notch1 or the amyloid precursor protein (APP), γ-Secretase acts as a central player in developmental processes and dysregulation of the enzyme can lead to the development of devastating diseases including acute T-cell leukemia (T-ALL) and Alzheimer’s disease (AD) [7, 8]. While SPPL3 controls the homeostasis of cellular glycosyltransferases, SPP is required for the propagation of several viruses including the Hepatitis C virus (HCV) [9] beyond its function in the degradation of signal peptides in the endoplasmic reticulum (ER) [10–12]. The rather ubiquitously expressed SPPL2a and SPPL2b proteins regulate immunological processes by impacting B cells and dendritic cells [13, 14]. Furthermore, they exert a protective function by controlling the atherogenic signaling of the lectin-like oxidized low-density lipoprotein receptor-1 (LOX-1) [15]. SPPL2c represents the most recently characterized member of the family. Surprisingly, this enzyme is exclusively expressed in round spermatids where it impacts sperm motility as well as acrosome formation [16, 17].

Initially, aspartyl I-CLIPs were considered to be rather unregulated enzymes based on the notion that they in many cases function in a sequential proteolytic process termed regulated intramembrane proteolysis (RIP). During RIP, a membrane spanning substrate is first processed by an ectodomain-removing protease (e.g., ADAM10/17) generating a membrane-bound stub which then can be cleared from the membrane by the consecutive action of a suitable intramembrane protease [18]. While ectodomain removal (also referred to as shedding) in most cases represents a highly controlled event, the involved I-CLIPs are thought to be constitutively active without any further regulation, thereby functioning as a kind of “proteasome of the membrane” [19]. This view was challenged by the finding that aspartyl intramembrane proteases can also directly cleave substrates with naturally short ectodomains without the requirement of any preceding processing [16, 17, 20, 21]. In these cases, their activity needs to be governed by distinct, yet poorly characterized mechanisms. One potential layer of control is provided by the interaction of I-CLIPs with accessory proteins that exert regulatory functions. While for γ-Secretase several interaction partners have been identified that in most of the cases stimulate the activity of the enzyme complex [22–25], only little is known about the regulation of SPPL proteases. We have recently identified a small testis-specific type II protein termed Frey regulator of sperm-oocyte fusion 1 (Frey1, also referred to as C11orf94 or 1700029I15Rik) as novel regulator of SPPL2c [26, 27]. Despite fulfilling all criteria for being a SPPL2c substrate, the intrinsically unstable Frey1 protein is stabilized within the catalytic center of the enzyme, thereby preventing SPPL2c-mediated intramembrane proteolysis of all tested substrates including Syntaxin-8, phospholamban (PLN), heme oxygenase 1 (HO-1) and stress-associated endoplasmic reticulum protein family member 2 (RAMP4-2) [26]. Though this establishes Frey1 as the first identified protein inhibitor of an aspartyl I-CLIP, the underlying molecular mechanism has remained elusive. Here, we have performed an extensive structure–function analysis of Frey1 aimed to decipher key components within this molecule crucial for its interaction with SPPL2c. Based on the unique inhibitory action of Frey1, we focused on the analysis of molecular features of Frey1 responsible for this aspect of the Frey1-SPPL2c interaction, while not addressing how stabilization of Frey1 by the protease is mediated. Employing this approach, we identify a motif within the Frey1 transmembrane domain (TMD), that is required for the inhibitory action of Frey1 on the dual aspartate catalytic center of SPPL2c. Notably, the inhibitory potential of the Frey1 TMD can be transplanted to the SPPL2c substrate PLN by the exchange of only five amino acids, thereby transforming it into an inhibitor of the enzyme. Furthermore, introduction of a critical motif within the N-terminus of the Frey1 transmembrane segment can even be utilized to generate Notch1-based inhibitors of γ-Secretase. Therefore, our study not only provides a detailed analysis of the molecular interactions underlying Frey1-mediated SPPL2c inhibition, but also opens the possibility of identifying unknown negative modulators of aspartic I-CLIPs in general.

## Results

### Frey1 inhibits SPPL2c by blocking substrate access to the catalytic center

**Fig. 1:**
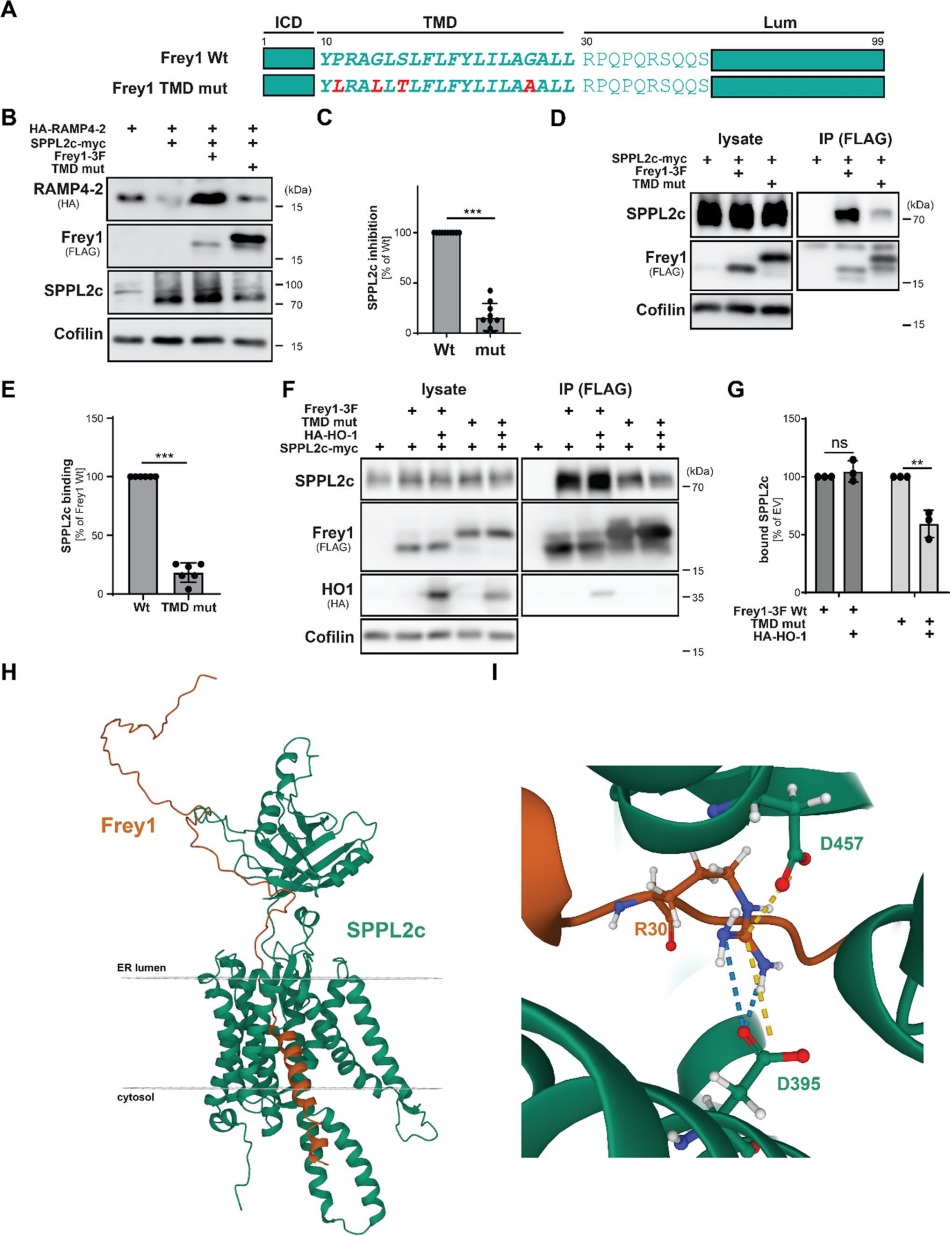
Frey1 is a competitive inhibitor of SPPL2c. **A** Sequence scheme of Frey1 and its TMD mutant (TMD mut). **B** HEK cells were transfected with the indicated constructs and monitored for SPPL2c-mediated cleavage of HA-RAMP4-2 by Western Blotting. **C** Quantification of **B**. *n* = 7. Unpaired two-tailed Student’s *t* test. **D** Interaction of wild-type Frey1-3xFLAG (Frey1-3F) and its TMD mutant with SPPL2c-myc was analyzed by co-immunoprecipitation experiments in transiently transfected HEK cells. **E** Quantification of **D**. Unpaired two-tailed Student’s *t* test. *n* = 6. **F** HEK cells were transiently transfected with the indicated constructs. Competition of wild-type Frey1-3xFLAG or its TMD mutant with the established SPPL2c-substrate HA-HO-1 for the catalytic center of SPPL2c was analyzed by co-immunoprecipitation experiments pulling on Frey1. **G** Quantification of **F**. *n* = 3. Unpaired two-tailed Student’s *t* test. **H** The structure of complexes formed by murine SPPL2c (green) and Frey1 (orange) was predicted using multimer versions of AlphaFold2. **I** Close-up of the interaction interface of the Frey1 JMD and the catalytically relevant aspartates in the active site of SPPL2c. Hydrogen bonds (blue dotted lines) and ionic interactions (yellow dotted lines) between the positively charged Frey1 R30 residue and the negatively charged D457 and D395 residues of SPPL2c are highlighted. *ns* not significant; **p ≤ 0.01; ***p ≤ 0.001

Even though the inhibitory function of Frey1 on SPPL2c is well documented [26], its precise mechanism has not been elucidated. Therefore, we first tested whether the small type II protein might bind to SPPL2c substrates, thereby preventing their recruitment to the active site of the protease. As demonstrated in Suppl. Figure 1A, we were unable to enrich the two established SPPL2c substrates HA-RAMP4-2 and HA-HO-1 above the level of background binding to the beads when precipitating 3xFLAG-tagged Frey1 (Frey1-3xFLAG) from transiently transfected HEK cells. Based on our published finding that binding of Frey1 to SPPL2c and its concomitant stabilization by the protease depend on the two catalytic aspartates of the enzyme [26], we speculated that Frey1 might directly engage the catalytic center of SPPL2c, thereby blocking access of substrate molecules. In line with this hypothesis and by contrast to Frey1-3xFLAG, HA-RAMP4-2 could not be co-precipitated with a C-terminally myc-tagged version of murine SPPL2c, suggesting that Frey1 and SPPL2c form a stable complex, while RAMP4-2 is efficiently cleaved by the protease only transiently occupying the catalytic site of the enzyme (Suppl. Figure 1B). In order to substantiate our findings, we aimed to design a mutant of Frey1 with reduced binding to SPPL2c which would still be recruited to the catalytic center yet lack any inhibitory function. If indeed Frey1 would act as a tightly binding competitive inhibitor, recruitment of this mutant to SPPL2c — but not wild-type Frey1 — should be reduced in presence of a substrate. Since helix-destabilizing residues have been suggested to play an essential role for recruitment of substrate molecules to aspartyl intramembrane proteases in general [28], we generated a mutant version of Frey1 in which several helix-destabilizing residues were replaced by helix-stabilizing amino acids (TMD mut, Fig. 1A). The mutated Frey1 variant largely co-localized with a co-expressed catalytically inactive variant of SPPL2c (D/A) indistinguishable from the wild-type protein, suggesting that potential differences could not be attributed to altered subcellular localization of this mutant (Suppl. Figure 2A). To test its inhibitory potential, we determined its effect on SPPL2c-mediated processing of HA-RAMP4-2 in HEK293T cells. In line with previous data, co-expression of SPPL2c-myc with HA-RAMP4-2 significantly decreased protein levels of the substrate. As observed for many other substrates of SPP/SPPL proteases [16, 17], the generated cleavage product could not be detected so that the evaluation of these assays was exclusively based on the detection of the protein levels of the full-length precursor. SPPL2c-mediated RAMP4-2 degradation was efficiently blocked by co-transfection with Frey1-3xFLAG. However, under the same conditions the TMD mutant was significantly less effective in inhibiting SPPL2c (Fig. 1B, C), while still able to bind to SPPL2c, albeit at much lower levels than the wild-type protein (Fig. 1D, E). Co-expression of SPPL2c together with the mutated Frey1 protein led to a stabilization of the mutant indistinguishable from wild-type Frey1 (Suppl. Figure 2B, C), suggesting that this variant is still recruited to the catalytic center of SPPL2c, but binds less efficiently. Having generated a mutant with the desired features, we employed either wild-type Frey1 or the described TMD mutant in a competitive co-immunoprecipitation experiment utilizing HO-1 as model substrate (Fig. 1F). While co-expression of HA-HO-1 had no effect on the amount of SPPL2c co-precipitating with wild-type Frey1, the presence of the substrate reduced the interaction of the TMD mutant with SPPL2c by about 50% (Fig. 1G). These data suggest a model in which Frey1 binds to the catalytic center of SPPL2c without being proteolyzed itself, thereby efficiently preventing proteolytic turnover of established SPPL2c substrates.

### Frey1 occupies the active site of SPPL2c

After obtaining experimental evidence for the molecular mechanism underlying Frey1-mediated SPPL2c inhibition, we aimed to scrutinize the molecular basis of this effect. For this purpose, we generated a high-confidence structural model using the Colab implementation of the AlphaFold2-multimer program [29, 30] based on the sequence of the murine proteins (Uniprot identifiers: Frey1: Q8CF31; SPPL2c: A2A6C4) in a template-free mode. As depicted in Fig. 1H, the top scoring complex structure predicts that Frey1 docks in the catalytic site of SPPL2c as expected from the experimentally generated data. Intriguingly, Frey1 occupied the same binding site as predicted for the established SPPL2c substrates Syntaxin-8 and RAMP4-2 again arguing for competition of Frey1 with substrate molecules for the active site of the enzyme (Suppl. Figure 2D). Even though these predictions represent speculative models, the binding conformation of Frey1 was similar to the cryo-EM structures of γ-Secretase complexed with either Notch1 or APP [31, 32] (Suppl. Figure 2E). In contrast to these experimental structures that capture only docked TMD fragments of the respective substrate proteins, both the Frey1 transmembrane segment (residues 10–29) and the extended chain of the juxtamembrane domain (JMD, residues 30–39) are involved in blocking access of substrate molecules to the catalytic center of SPPL2c, enabled by the sequestration of the Frey1 TMD from the membrane (Fig. 1H). In agreement with previous findings that the two catalytic aspartates (D395 and D457) of SPPL2c are required for efficient interaction with Frey1 [26], these two residues are closely positioned against the oppositely charged R30 residue of Frey1 (Fig. 1I). The triangular geometry of these charged residues is critically stabilized by electrostatic interactions between R30 of Frey1 and the two catalytic aspartates of SPPL2c, as well as potential hydrogen bonds between R30 and D395. In short, instead of cleaving the captured Frey1 chain, the bound inhibitor freezes the catalytic constellation of the protease with an electrostatic lock. Beyond the interactions of the Frey1 TMD and JMD with the catalytic center of SPPL2c, the AlphaFold2-generated model also predicts that a portion of the Frey1 luminal chain (residues 38–54) docks along a surface groove on the N-terminus of SPPL2c, making a range of additional stabilizing contacts (Fig. 1H).

### The N-terminal part of the Frey1 TMD is essential for inhibition of SPPL2c

Since the predicted structure of the murine SPPL2c/Frey1 complex suggested an involvement of both the trans- and juxtamembrane domains of the Frey1 molecule in the inhibition of SPPL2c, we next investigated whether these elements might contribute to this effect. Therefore, we employed a detailed mutational analysis of Frey1 utilizing chimeras with the established SPPL2c substrate phospholamban (PLN). This choice was based on several considerations. On the one hand, PLN and Frey1 share important features including type II topology, the length of their predicted transmembrane helix and ER localization. Additionally, both proteins are recruited to the catalytic center of SPPL2c [17, 26]. However, while this leads to productive proteolytic cleavage of PLN, recruitment of Frey1 facilitates its stabilization, thereby blocking SPPL2c-mediated proteolysis [26]. These opposing effects of SPPL2c on Frey1 and PLN make both proteins an ideal pair for generation of chimeric proteins to assess which parts of Frey1 contribute to its inhibitory capacity toward SPPL2c. The co-localization of all generated mutants of Frey1 and PLN with an inactive (D/A) variant of SPPL2c was additionally monitored by indirect immunofluorescence. As depicted in Suppl. Figures 3–4, the subcellular localization of all tested mutants overlapped with that of the SPPL2c D/A mutant excluding mistrafficking as causative factor for potential differences in the inhibitory capacity of the generated Frey1 or PLN mutants.

**Fig. 2:**
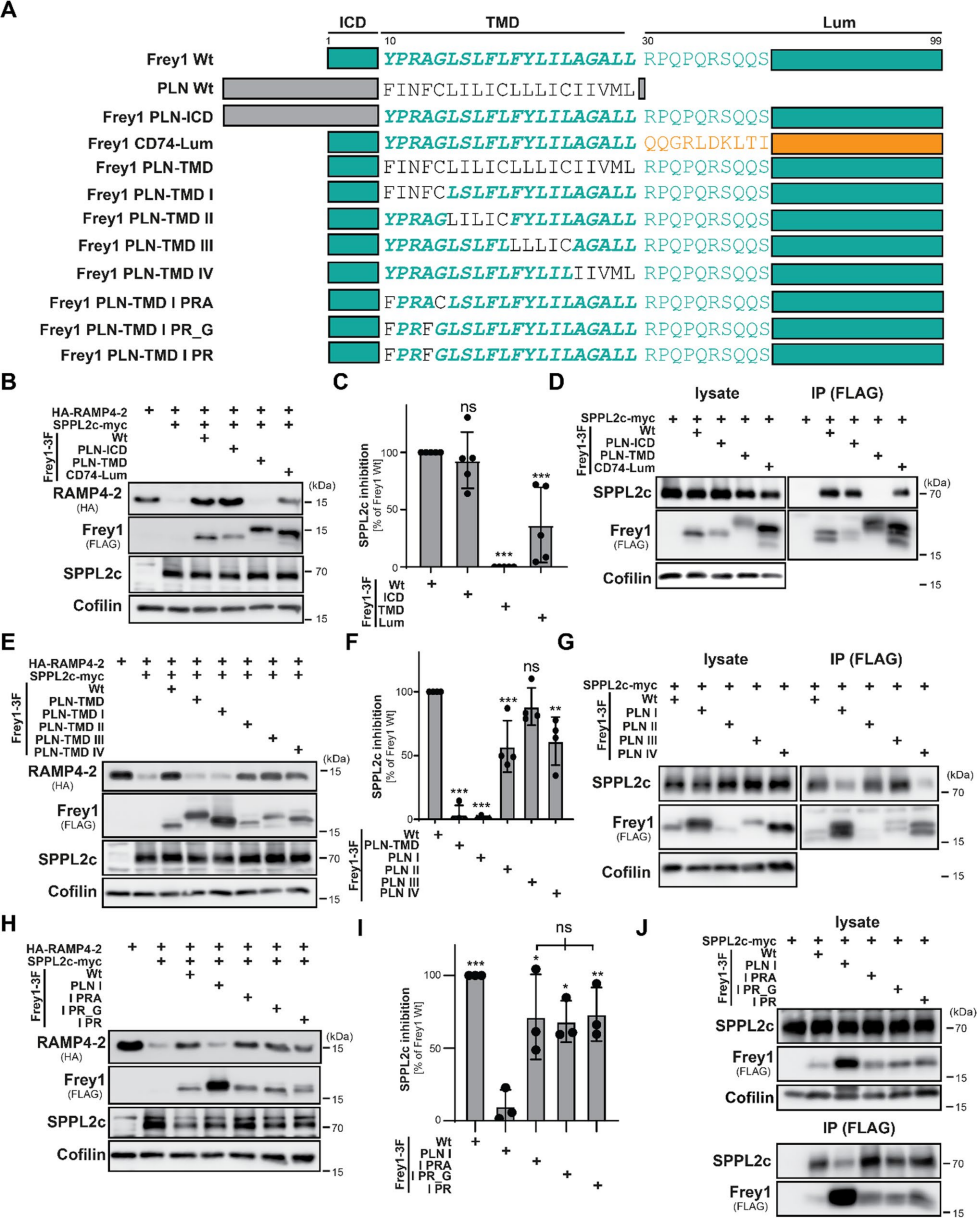
A PR motif within the Frey1 TMD is essential for SPPL2c inhibition. **A** Overview of the Frey1 variants employed in this figure. **B** HEK cells were transiently transfected with the indicated constructs and monitored for inhibition of SPPL2c-mediated RAMP4-2 proteolysis by Western Blotting. **C** Quantification of **B**, *n* = 5. **D** Physical interaction between SPPL2c-myc and each Frey1-3xFlag mutant was validated in HEK cells by Western Blot of immunoprecipitated proteins using a FLAG-targeting antibody. **E** SPPL2c inhibition assay using Frey1 transmembrane domain chimeras in which blocks of five amino acids were exchanged by the corresponding residues of the PLN TMD. **F** Quantification of **E**, *n* = 4. **G** Anti-Flag immunoprecipitation assay using Frey1 transmembrane domain mutants. **H** The inhibition capacity of Frey1 PLN-TMD segment I mutants in which several amino acids were remutated simultaneously was monitored by Western Blot analysis of HEK cells transiently transfected with the indicated constructs. **I** Quantification of **H**, *n* = 3. One-way ANOVA followed by Tukey’s multiple comparisons test. **J** Binding of Frey1 PLN-TMD I mutants with double or triple reinsertions to SPPL2c-myc was analyzed by co-immunoprecipitation using FLAG-directed antibodies together with lysates of transfected HEK cells. Statistical analyses, in **C**, **F** and **I** were performed by a one-way ANOVA with Dunnett’s post hoc testing. *ns* not significant; *p ≤ 0.05; **p ≤ 0.01; ***p ≤ 0.001

We generated mutants in which either the short intracellular domain (ICD) or hydrophobic transmembrane segment of Frey1 was exchanged with the respective parts of PLN (Fig. 2A). Since PLN essentially lacks a luminal domain, we replaced this section of Frey1 with the first 70 aa of the corresponding segment of CD74 (Frey1 CD74-Lum, Fig. 2A), an established substrate of SPPL2a [13]. The inhibitory potential of these chimeras on SPPL2c was then tested employing the HA-RAMP4-2 cleavage assay described above. Inhibition of SPPL2c was not, or only moderately, altered by exchange of the cytosolic part or luminal domain of Frey1. However, mutant Frey1 equipped with the transmembrane segment of PLN was completely incapable of preventing SPPL2c-mediated intramembrane proteolysis, suggesting this part of Frey1 has a dominant role for SPPL2c inhibition (Fig. 2B, C). These findings were further substantiated by the analysis of the interaction of the chimeric proteins with SPPL2c. While SPPL2c could be efficiently co-precipitated with wild-type Frey1 and Frey1 PLN-ICD, exchange of the Frey1 luminal domain to a corresponding stretch of CD74 reduced the binding of mutant to SPPL2c to about 20% of the wild-type protein when normalized to the expression level of these variants. By contrast, swapping the transmembrane helices of Frey1 with that of PLN completely abolished binding under the tested conditions, thereby explaining the lack of inhibition observed for this mutant again pointing to a dominant role of the Frey1 transmembrane segment for interaction with and inhibition of SPPL2c (Fig. 2D, quantification in Suppl. Figure 5A).

Despite its moderate effect on binding and inhibition, we next scrutinized the part of the Frey1 luminal domain involved in SPPL2c inhibition. Since the generated AlphaFold2-multimer model suggested docking of the extended JMD to the luminal end of the catalytic center of SPPL2c by R30 (Fig. 1H, I), we concentrated on the relevance of this residue for Frey1-mediated SPPL2c inhibition. Based on the crucial role of the positive charge at R30 for the Frey1–SPPL2c interaction implied by the predicted structure of the Frey1/SPPL2c complex, we generated two mutants in which R30 was exchanged by negatively charged D or E residues. As control, R30 was replaced by lysine preserving the positive charge at this position. In agreement with the suggestions from the AlphaFold2 model, introduction of a negative charge at position 30 of Frey1 resulted in an almost complete loss of the inhibitory capacity of the small type II protein (Suppl. Figure 5B, C). By contrast, preserving the positive charge of the wild-type protein at this position by an arginine/lysine exchange did not significantly alter the function of Frey1. In line with these findings, the R30D and R30E mutations presented impaired binding to SPPL2c, though to different extent and not as clearly as the Frey1 TMD mutant (Fig. 2B, C). The R30K variant did not reveal an altered interaction with the protease if compared to the wild-type protein (Suppl. Figure 5D, E) highlighting the relevance of a positive charge at position 30 of the Frey1 protein for interaction with SPPL2c.

Having established a role for R30 in the regulatory interaction of Frey1 with SPPL2c, we focused on delineating the exact amino acids of the Frey1 transmembrane segment involved in binding and inhibition of this protease since this core region showed the greatest sensitivity to mutational change in the initial screening experiment (Fig. 2B, C). Therefore, we generated Frey1 TMD chimeras in which blocks of five amino acids were exchanged by those of the corresponding section of PLN (I, II, III, IV, scheme in Fig. 2A) and employed them in our RAMP4-2 cleavage assay. Strikingly, swapping the more C-terminal parts of the transmembrane segment only mildly altered (II and IV) or did not (III) affect SPPL2c-mediated RAMP4-2 cleavage. By contrast, alteration of the first five residues of the Frey1 TMD (I) rendered the molecule completely inactive in terms of SPPL2c inhibition indistinguishable from the mutant in which the full domain was exchanged (Fig. 2E, F). Again, this was connected to a clear decrease in interaction with SPPL2c (Fig. 2G, quantification in Suppl. Figure 5F). Surprisingly, the exchange of the five C-terminal residues of the Frey1 TMD by those from the corresponding segment of PLN (IV) heavily decreased binding to SPPL2c comparable to the TMD I mutant albeit having a much smaller impact on the inhibitory capacity of Frey1 (Fig. 2E, F). Since binding of Frey1 is required, but not sufficient for preventing SPPL2c-mediated proteolysis, these findings point to an inhibitory motif in the first five amino acids of its transmembrane segment.

### An N-terminal PR sequence in the Frey1 TMD is required for inhibition of SPPL2c

To investigate the contribution of individual amino acids in the YPRAG sequence of Frey1 (residues 10–14) for SPPL2c inhibition, we re-introduced each of the initial five residues of the Frey1 transmembrane segment individually into the Frey1 PLN-TMD I construct (scheme in Suppl. Figure 6A) and evaluated binding and inhibition of SPPL2c by these mutants. Re-introduction of P11 partially restored binding to SPPL2c, which to a lower degree was also observed upon restoration of R12 and G14 residues (Suppl. Figure 6B, C). In line with these findings, with exception of the PLN-TMD I I/P mutant that caused a weak but significant inhibition of SPPL2c, none of the other mutants affected SPPL2c-dependent processing of RAMP4-2 (Suppl. Figure 6D, E) suggesting that a combination of different residues within the YPRAG sequence of the Frey1 transmembrane helix is required for inhibition. Indeed, combinational reinsertion of PRA and PR_G residues as well as the PR sequence shared by these two mutants into Frey1 PLN-TMD I largely restored the inhibitory potential of the mutant proteins (Fig. 2H, I) as well as binding to SPPL2c (Fig. 2J, quantification in Suppl. Figure 6F).

### The inhibitory features of the Frey1 TMD can be transplanted to other type II proteins

**Fig. 3:**
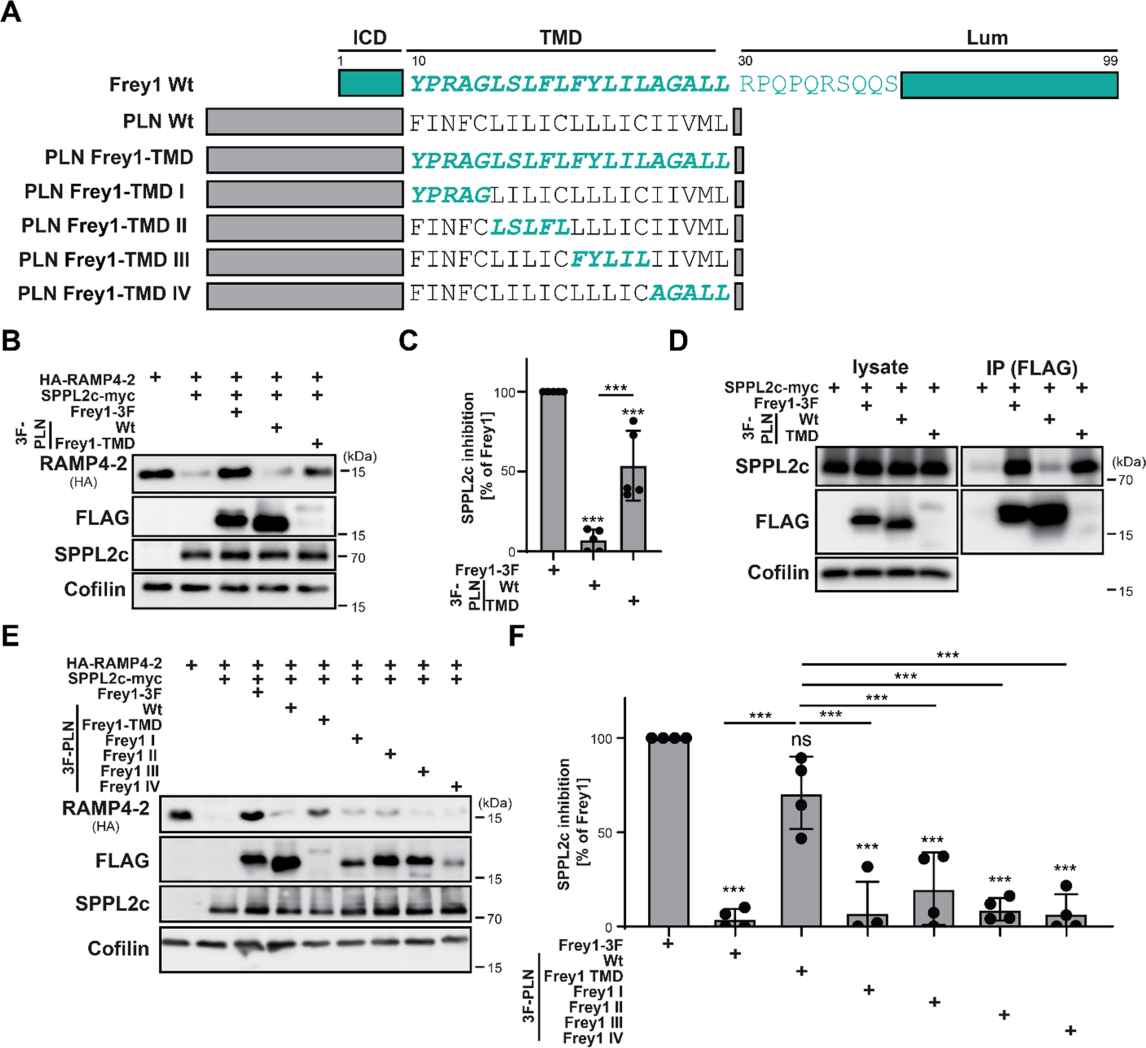
The inhibitory features of the Frey1 TMD can be explanted to PLN. **A** Schematic representation of PLN-Frey1 chimeras employed in this figure. **B** HEK cells transiently transfected with the indicated constructs were lysed and analyzed by Western Blotting for SPPL2c inhibition assay by the different constructs. **C** Quantification of **B**, *n* = 5. One-way ANOVA followed by Tukey’s multiple comparisons test. **D** Western Blot of co-immunoprecipitated proteins using anti-FLAG versus total lysates from HEK cells transiently transfected with indicated constructs. **E** SPPL2c-mediated processing of HA-RAMP4-2 in presence of PLN Frey1-TMD chimeras was monitored by Western Blotting of lysates from transfected HEK cells. **F** Quantification of **E**, *n* = 4. One-way ANOVA followed by Tukey’s multiple comparisons test. *ns* not significant; ***p ≤ 0.001

Having identified the PR motif within the N-terminus of the Frey1 transmembrane segment as key inhibitory sequence, we wondered whether this motif could be transplanted into other substrate proteins, thereby generating novel inhibitors of SPPL2c. To test this hypothesis, we reversed our initial approach and exchanged the full TMD of PLN by that of Frey1 (PLN Frey1-TMD, scheme in Fig. 3A). Interestingly, the transplanted Frey1 transmembrane segment conferred the intrinsically low stability of Frey1 to the host protein (Fig. 3B). In line with this, the PLN Frey1-TMD mutant in contrast to wild-type PLN significantly inhibited SPPL2c-mediated processing of RAMP4-2 (Fig. 3B, C). Furthermore, insertion of the Frey1 TMD enabled PLN to bind SPPL2c at levels comparable to Frey1 (Fig. 3D). When normalized to the amount of immunoprecipitated protein levels, the chimeric PLN mutant interacted even more efficiently with SPPL2c than wild-type Frey1 (Suppl. Figure 7A). To validate that this was caused by the introduction of the PR motif, following our initial approach we divided the PLN transmembrane helix into five amino acid blocks and swapped them with the corresponding stretches of the Frey1 protein (PLN Frey1-TMD I/II/III/IV, scheme in Fig. 3A). However, insertion of none of these blocks, not even section I carrying the previously identified inhibitory PR motif, recapitulated the inhibition of SPPL2c-mediated HA-RAMP4-2 proteolysis as observed upon expression of PLN Frey1-TMD (Fig. 3E, F).

**Fig. 4:**
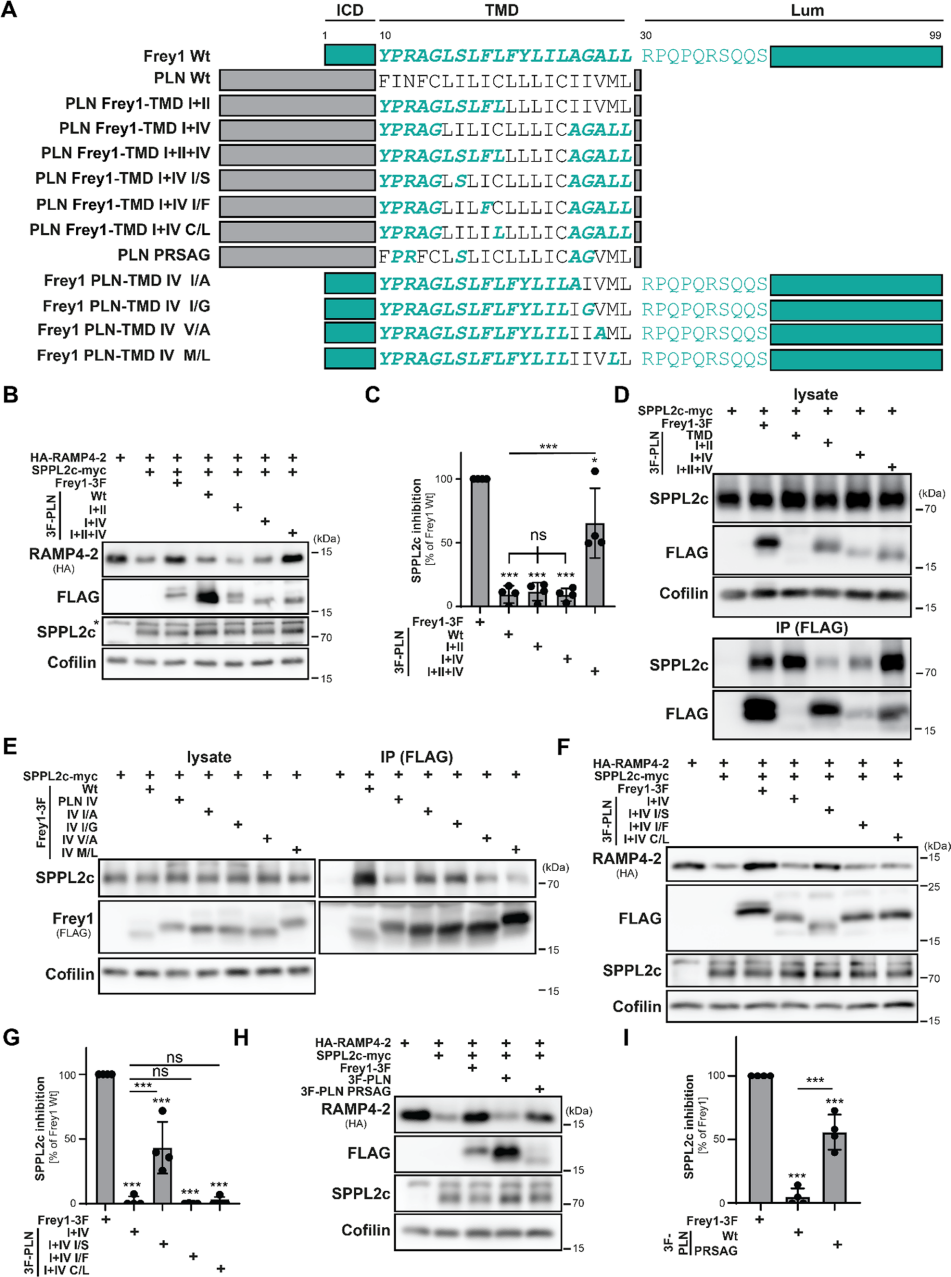
Exchange of only five residues transforms PLN into a SPPL2c inhibitor. **A** Schematic representation of constructs employed in this figure. **B** Inhibition of SPPL2c activity was monitored by Western Blotting of lysates of transfected HEK cells based on RAMP4-2 degradation in presence of PLN variants with insertion of multiple blocks of five amino acids from the Frey1 TMD. **C** Quantification of **B**, *n* = 4. One-way ANOVA followed by Tukey’s multiple comparisons test. **D** Western Blot of total lysates and anti-FLAG immunoprecipitated proteins from HEK cells transiently transfected with the indicated mutants. **E** Binding of PLN Frey1-TMD IV mutants in which individual residues were re-mutated to the Frey1 sequence was analyzed by co-immunoprecipitation experiments employing transfected HEK cells and anti-FLAG. **F** Relevance of the Frey1 residues within section II of its TMD for SPPL2c inhibition was investigated by transient transfection of HEK cells and subsequent Western Blotting. **G** Quantification of **F**, *n* = 4. One-way ANOVA followed by Tukey’s multiple comparisons test. **H** SPPL2c inhibition assay based on RAMP4-2 degradation in presence of a PLN mutant containing the five residues of Frey1 identified to mediate SPPL2c inhibition. **I** Quantification of **H**, *n* = 4. One-way ANOVA followed by Tukey’s multiple comparisons test. *ns* not significant ***p ≤ 0.001

This suggested that the presence of the PR motif alone is not sufficient for efficient binding to the catalytic center and concomitant inhibition of SPPL2c in the context of the PLN TMD. To identify the other yet unknown factors within the Frey1 transmembrane segment required for SPPL2c inhibition, we generated PLN mutants in which several blocks of the Frey1 TMD were inserted simultaneously (scheme in Fig. 4A). Based on our initial findings from the mutational analysis of Frey1, we focused on blocks I, II and IV since exchange of segment III did neither impact the inhibitory potential of the protein nor its ability to interact with SPPL2c (Fig. 2E–G). As shown in Fig. 4B and quantified in Fig. 4C, inhibition of SPPL2c was only achieved upon simultaneous co-insertion of Frey1 TMD blocks I, II and IV into PLN highlighting the contribution of critical residues in both segment II and IV in addition to the PR motif in segment I. In agreement with these data, binding of Frey1-PLN TMD to SPPL2c was only restored to a level comparable to that of the Frey1 PLN-TMD mutant when the same three segments were reinserted into this mutant (Fig. 4D, quantified in Suppl. Figure 7B). To identify the critical residues in segment IV of the Frey1 transmembrane segment (which had already been shown to play a crucial role for the interaction of Frey1 with SPPL2c, Fig. 2G), we mutated individual amino acids in the Frey1 PLN-TMD IV backbone to the original residues of the Frey1 transmembrane helix and analyzed the interaction of the resulting mutants with SPPL2c by co-immunoprecipitation experiments (scheme in Fig. 4A). Even though none of the generated mutants fully restored binding to SPPL2c to the level of the wild-type protein, re-insertion of A25 and G26 into the Frey1 PLN-TMD IV mutant increased co-precipitation of SPPL2c (Fig. 4E, quantified in Suppl. Figure 7C). We next sought to scrutinize the critical residues within segment II of the Frey1 TMD with a different strategy, since the initial exchange of this block in the context of Frey1 did neither clearly impact SPPL2c inhibition nor binding (Fig. 2E–G). Based on the notion that the PLN Frey1-TMD I + II + IV mutant significantly inhibited HA-RAMP4-2 processing by SPPL2c, while PLN Frey1-TMD I + IV did not (Fig. 4B, C), we individually inserted the Frey1 transmembrane segment II-specific residues into the PLN Frey-TMD I + IV backbone and monitored the resulting mutants for their inhibition of SPPL2c-mediated proteolysis (scheme in Fig. 4A). While insertion of the F18 or L19 residues of Frey1 did not cause any detectable inhibition of SPPL2c, this was at least partially observed in the presence of PLN Frey1-TMD I + IV I/S mutant (Fig. 4F, G) highlighting the importance of S16 for inhibition of SPPL2c.

Our findings indicate that residues S16, A25 and G26, added to the PR motif (residues 11–12) in the N-terminus of the Frey1 transmembrane segment, are required for optimal transfer of the inhibitory function of the Frey1 TMD to PLN. To validate this experimentally, we generated a mutant version of PLN in which only these residues of Frey1 were introduced into the corresponding positions of the PLN membrane anchor (PLN PRSAG, scheme in Fig. 4A) and tested it in our HA-RAMP4-2 cleavage assay. In agreement with our hypothesis and in contrast to wild-type PLN, the PLN PRSAG mutant inhibited SPPL2c-mediated processing of the reporter protein by about 50% of the level of wild-type Frey1, despite being expressed at a lower level (Fig. 4H, I). Similarly, the PRSAG mutant bound to SPPL2c more efficiently than wild-type PLN (Suppl. Figure 7D, E) highlighting the relevance of these residues not only for SPPL2c inhibition but also binding. Based on these findings, we suggest a model in which residues mapping to the N- and the C-termini of the Frey1 TMD as well as R30 in the juxtamembrane domain of this protein are essential for its interplay with SPPL2c (Suppl. Figure 7). Intriguingly, while A25 and G26 principally impact the binding of Frey1 to SPPL2c rather than inhibition of the enzyme, the P11 residue is essential for both aspects of the Frey1–SPPL2c interaction. R12 seems to be particularly relevant for inhibition while affecting binding to the protease to a much lesser degree. This suggests a model in which the helix-breaking properties of P12 allow easy accession of the positive charge at R12 to the catalytically relevant aspartates of the intramembrane protease, leading to an electrostatic blockade in SPPL2c enzymatic activity.

### The findings from Frey1 and SPPL2c can be transferred to γ-secretase

**Fig. 5:**
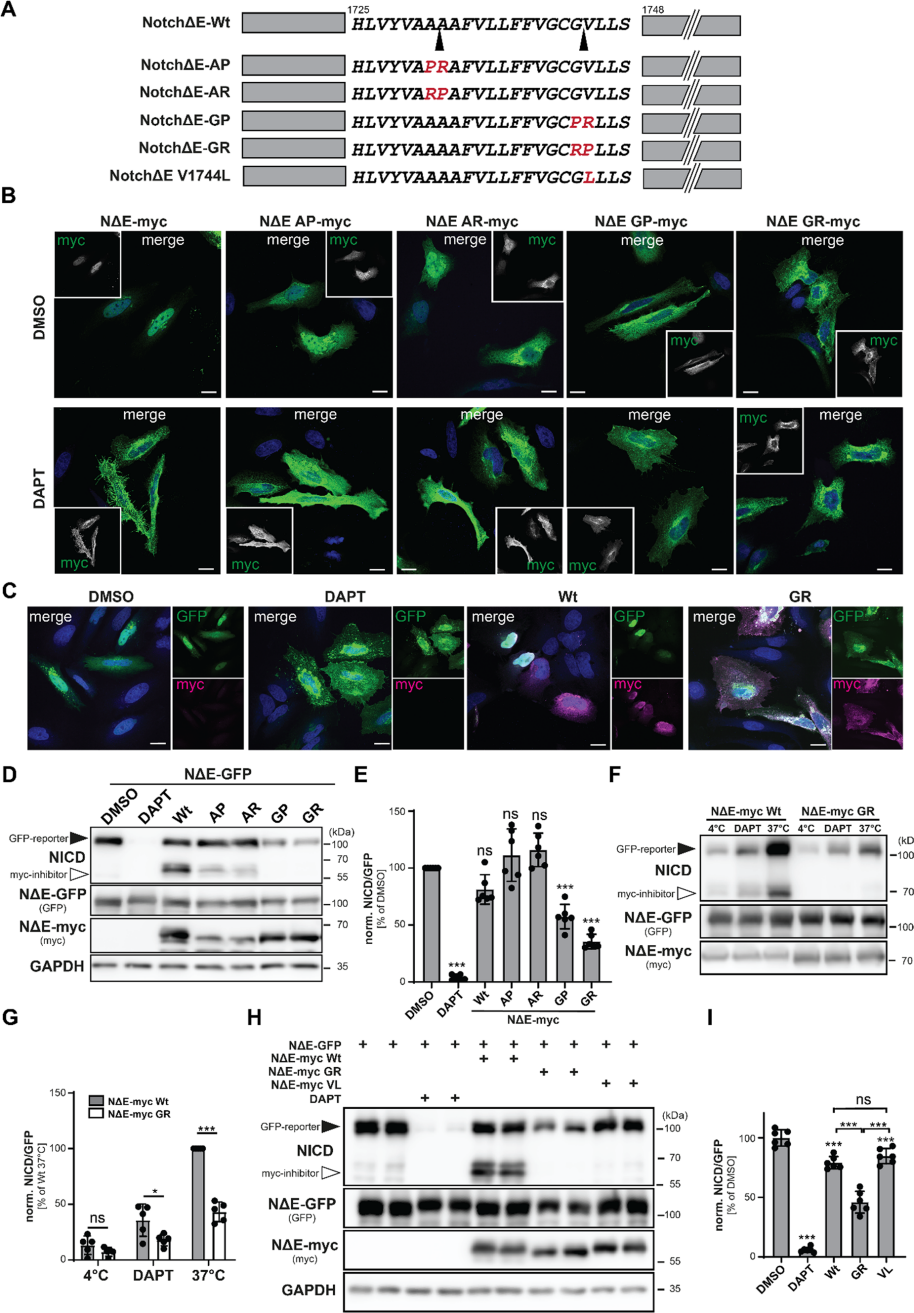
The findings from Frey1 and SPPL2c can be transferred to γ-Secretase. **A** Scheme of the NΔE-mutants employed for the generation of data presented in this figure. Cleavage sites within murine Notch1 are highlighted with arrow heads. The numbers above the scheme indicate the position of the TMD in the wild-type Notch1 protein to allow better comparison to other published data. **B** HeLa cells were transiently transfected with the indicated constructs and treated with DMSO or 10 µM DAPT overnight. After fixation with 4% PFA, cells were subjected to indirect immunofluorescence analysis using anti-myc. Nuclei were visualized with DAPI. Scale bars, 20 µm. **C** Processing of the NΔE-eGFP reporter was analyzed by indirect immunofluorescence in Hela cells transiently transfected with the indicated constructs. As control, a subset of cells was treated with 10 µM DAPT for 16 h. **D** HEK cells were transiently transfected with NΔE-eGFP and either wild-type NΔE-myc or its indicated mutants. Additionally, some cells were treated with 10 µM DAPT for 16 h to inhibit γ-Secretase pharmacologically. Processing of NΔE-eGFP was subsequently analyzed by Western Blotting using the indicated antibodies. **E** Quantification of **D**, *n* = 6. One-way ANOVA with Dunnett’s multiple comparisons test. **F** CHAPSO-solubilized membrane preparations of HEK cells overexpressing NΔE-eGFP together with either NΔE-myc Wt or its GR mutant were incubated for 4 h at 37 °C. Subsequently, generation of the NICD was monitored by Western Blotting using an antibody specifically detecting this cleavage fragment. To control for γ-Secretase activity underlying the observed NICD generation, membrane preparations were incubated for the same time either at 4 °C or in the presence of 10 µM DAPT to interfere with the activity of the enzyme complex. **G** Quantification of **F**. For statistical analysis, for each condition an unpaired two-tailed Student’s *t* test was performed. **H** The NΔE-eGFP cleavage assay described in **D** was repeated employing the uncleaved NΔE-myc V1744L mutant as additional control. **I** Quantification of **H**. One-way ANOVA with Tukey’s multiple comparisons test. *ns* not significant; *p ≤ 0.05; ***p ≤ 0.001

Despite major differences in substrate selectivity and membrane orientation, all aspartyl I-CLIPs identified to date share the same catalytic mechanism involving critical aspartates within TMD6 and TMD7 of the shared enzyme fold and helical unwinding of the substrate in the vicinity of the catalytic site [31–34]. Due to this universal mechanistic blueprint, we speculated that the combination of the PR motif crucial for Frey1-mediated inhibition of SPPL2c might be applied to other protease-substrate pairs to generate potent substrate-based inhibitors of these enzymes. To assess this possibility, we focused on γ-Secretase, which plays a central role in several (patho-)physiological processes including the development of T-ALL as well as AD [8, 35]. As a model substrate we selected Notch1, which has known γ-Secretase cleavage sites and detailed structural data available with a 2.6 Å resolution cryo-EM of the human Notch1 transmembrane segment bound to the catalytic center of presenilin1 [31]. Since γ-Secretase-mediated processing of Notch1 requires the removal of its N-terminal ectodomain [36], we utilized a truncated version of murine Notch1 (NΔE) that lacks this part of the protein and therefore can be directly cleaved by γ-Secretase without any preceding activating step [37, 38] as starting point for our mutational analysis. As contact of the PR motif to the catalytically relevant aspartates within the presenilins is required for inhibition, so we replaced the P1 and P1′ residues flanking the major cleavage sites after A1731 (NΔE-AP) and G1743 (NΔE-GP) with the inhibitory PR motif. Additionally, since presenilins have an inverted membrane topology as compared to SPPL2c, we generated mutants in which the P1 and P1′ residues were replaced by an RP combination (NΔE-AR or -GR, respectively) (Fig. 5A). We first tested whether the resulting mutants could still be cleaved by γ-Secretase since this would exclude them as potential inhibitors of this enzyme. Myc-tagged wild-type NΔE and the mutants in which the cleavage site following A1731 was altered were efficiently processed by γ-Secretase as judged from nuclear localization of the liberated Notch intracellular domain (NICD) upon expression of these constructs in HeLa cells. However, in both mutants a significant ER localization was observed indicating impaired folding or transport of the corresponding proteins. The requirement of γ-Secretase activity for the observed nuclear localization of the Notch1 variants was further validated by treatment with the specific γ-Secretase inhibitor DAPT which shifted the Notch1 staining to the plasma membrane. By contrast, modifying the cleavage site at G1744 with either the PR or the inverted RP motif largely (GP mutant) or almost completely (GR mutant) abolished γ-Secretase-mediated processing (Fig. 5B, quantification in Suppl. Figure 8A). However, NΔE-GP-myc was largely retained in the ER explaining the reduced NICD generation from this construct. By contrast and despite some minor ER localization, the NΔE-myc GR mutant largely accumulated at the plasma membrane as expected from an uncleavable mutant. Concomitantly, γ-Secretase inhibition by DAPT did not influence the localization of this Notch1 mutant. Next, we analyzed the NΔE-myc mutants for their inhibitory potential on γ-Secretase. For this purpose, we utilized NΔE-eGFP as reporter molecule, which as described above is constitutively processed by γ-Secretase to the resulting NICD-eGFP fragment that translocates to the nucleus [37]. We co-expressed either wild-type NΔE-myc or the equivalently tagged mutants together with NΔE-eGFP in HeLa cells and analyzed them by indirect immunofluorescence for the nuclear presence of NICD-eGFP. Inhibition of NΔE-eGFP processing was compared to cells treated with the established γ-Secretase inhibitor DAPT as a control. While in DMSO-treated as well as in wild-type NΔE-myc co-transfected control cells the reporter was efficiently proteolyzed by γ-Secretase and therefore largely detected in the nucleus, co-transfection of NΔE-myc GR caused a clear reduction of the nuclear GFP signal at the same time leading to an accumulation of the uncleaved precursor at the plasma membrane similar to the situation of cells treated with DAPT (Fig. 5C). While this was observed on a lower level also for the NΔE-myc GP mutant, in line with their cleavability by γ-Secretase NΔE-myc AP and -AR did not prevent generation and nuclear translocation of NICD-eGFP (Suppl. Figure 8B, quantification for all constructs in Suppl. Figure 8C). To assess γ-Secretase inhibition more quantitatively, fragment levels generated from NΔE-eGFP were analyzed in an identical setting by Western Blotting in transfected HEK cells. As shown in Fig. 5D and quantified in Fig. 5E, only co-expression of NΔE-myc GP and NΔE-myc GR caused a significant decrease of NICD-eGFP generation, which was more pronounced in case of the NΔE-myc GR variant. The same effect could be observed for γ-Secretase-dependent processing of an HA-tagged variant of the C99 fragment of human APP (hC99-HA, Suppl. Figure 8D, E), demonstrating that the observed inhibitory function of the NΔE-myc GR construct was not substrate-specific. Even though in these cells also co-expression of wild-type NΔE-myc caused a significantly reduced degradation of hC99-HA most likely due to the fact that Notch1 and APP compete for the catalytic center of γ-Secretase [39], the effect observed upon co-transfection with the NΔE-myc GR construct was significantly stronger although this construct was expressed at lower levels than the wild type (Suppl. Figure 8D, E). Inhibition of γ-Secretase by this mutant could additionally be validated employing γ-Secretase in vitro assays based on membrane preparations of HEK cells transiently overexpressing either NΔE-eGFP together with NΔE-myc Wt or its GR variant (Fig. 5F, G). Similar results were obtained for hC99-HA as substrate in the same assay system (Suppl. Figure 8F, G). To further demonstrate that γ-Secretase inhibition was indeed a specific feature of the generated GR mutant and did not represent an intrinsic feature of any non-proteolyzed Notch1 variant, we introduced the V1744L mutation into NΔE-myc as control. This mutation has been reported to be cleaved by γ-Secretase to negligible degree [40]. Indeed, NΔE-myc V1744L predominantly localized to the plasma membrane when overexpressed in HeLa cells both in DMSO- and DAPT-treated conditions (Suppl. Figure 8H, I) validating the impaired cleavability of this mutant by γ-Secretase. However, unlike the GR mutant, NΔE-myc V1744L did not reduce NΔE-eGFP proteolysis both in Western Blot (Fig. 5H, I) and immunofluorescence analysis (Suppl. Figure 8 J, K), thereby strongly suggesting that the inhibitory action of the PR/RP substrate mutants does not solely rely on competitive inhibition but instead might be based on an electrostatic block within the catalytic site of the respective aspartyl intramembrane protease.

## Discussion

Even though failure to inhibit SPPL2c is not causative for the infertility of Frey1-deficient male mice [26, 27], our detailed molecular analysis of the interaction of these two molecules has provided deep insights into the mechanistic action of SPPL2 proteases and of physiological regulation of aspartyl I-CLIPs in general.

Albeit Frey1 inhibits SPPL2c activity rather than being proteolyzed by this enzyme itself, our findings for the SPPL2c/Frey1 interaction agree surprisingly well with other data regarding the interaction of SPPL2 proteases with their substrates. The AlphaFold2-multimer model of the Frey1–SPPL2c complex provides a confident structural foundation for discussion and experiments; hence, it should serve as a springboard guiding biophysical studies [41] to capture the precise details of the SPPL2c–Frey1 interaction to build on the structural knowledge from other aspartyl I-CLIP complexes. Little is known about the exact interactions of SPPL2 family proteases with their substrates. Intramembrane proteolysis critically depends on (1) entry and proper docking of the substrate TMD to the protease, (2) local unwinding of the transmembrane helix and juxtamembrane segment in the solvent-accessible environment of the active site, (3) pinning of the JMD N-terminus to the protease by forming a compact β-sheet structure, and (4) presentation of the cleavage site—typically at the transition from transmembrane helix to unwound JMD—to the catalytic Asp dyad in a precise geometry [34, 42–44]. However, while evaluation of substrate mutants in previous studies has mainly focused on their general cleavability, we further bolstered our analysis by the inclusion of AI-based structure predictions and co-immunoprecipitation assay-based interaction data that complete our understanding of the ongoing processes. We propose a model in which Frey1 initially binds to SPPL2c by TMD docking to the binding cleft and JMD incorporation to the small β-sheet in the protease active site cavity that fixes and presents the substrate chain to the catalytic Asp residues (Suppl. Figure 7F). Intriguingly, this appears to be dependent on a positive charge at position 30 within the luminal part of Frey1. Indeed, JMDs play an essential role in substrate processing both for SPPL2 proteases but also γ-Secretase [43, 45, 46], suggesting that the presence of positive charges within these segments might be a common, even though certainly not universal theme for aspartyl I-CLIP substrates. This notion is further supported by the capability of SPP/SPPL2 proteases to process type IV proteins that essentially lack a luminal/extracellular domain [16, 17, 20, 47] which therefore cannot be essential for initiation of substrate-enzyme contacts.

Our data indicate that P11 and R12 in the Frey1 transmembrane segment are essential for the inhibition of SPPL2c-mediated intramembrane proteolysis by electrostatically blocking the catalytically relevant aspartates within the active site of the enzyme. As an alternative explanation, Frey1 could represent an explicitly slowly processed substrate of SPPL2c that prevents proteolysis of other type II-oriented TMDs in a purely competitive manner. Indeed, many mutants presented in this study that blocked SPPL2c-mediated cleavage of RAMP4-2 appeared to be expressed at rather low levels when co-expressed with SPPL2c which might indicate proteolytic turnover of these Frey1 variants. This hypothesis is strengthened by reports of competitive inhibition of γ-Secretase-dependent processing of APP and Notch1 [48, 49]. In fact, APP C99 and constitutively cleaved versions of Notch prevent cleavage of the other substrate in a dose-dependent manner [39] This could also be observed in this study, where co-expression of NΔE-myc significantly increased levels of hC99-HA in comparison with control cells (Suppl. Figure 8D, E). However, there are several arguments that cannot easily be brought in congruence with this hypothesis. First, as demonstrated before, Frey1 specifically interacts only with wild-type SPPL2c while binding much less efficiently to inactive variants in which the catalytically relevant aspartates are mutated [26]. By contrast, substrates of SPPL2 proteases behave in an opposite manner, co-precipitating with aspartate mutants of SPP/SPPL proteases but not with the corresponding wild-type proteins [50, 51]. In line with this, loss of SPPL2c causes a drop in Frey1 protein levels in the murine testis under physiological conditions [26], while even in the case of a particularly slowly processed substrate one would expect an accumulation upon loss of the degrading protease. Additionally, AlphaFold2 predictions as well as competitive co-immunoprecipitation experiments presented in this study suggest that Frey1 occupies the catalytic center of SPPL2c (Fig. 1, Suppl. Figure 2). Furthermore, the generic PLN Frey1-TMD construct inhibited SPPL2c efficiently despite being expressed at much lower levels than wild-type Frey1, while high amounts of PLN did not significantly interfere with SPPL2c-mediated RAMP4-2 cleavage (Fig. 3B, C). Similarly, NΔE-myc GR carrying a RP insertion at the major γ-Secretase cleavage site of the receptor inhibited turnover of both, NΔE-eGFP and hC99-HA, more efficiently than the corresponding wild-type protein even though being expressed at lower levels. This was valid both for cell-based but also in vitro assays that are widely utilized for the assessment of γ-Secretase activity. These data clearly point to a crucial role of the RP/PR motifs for efficient inhibition. Perhaps the strongest evidence for the hypothesis that the PR/RP motif does not only generate uncleavable mutants which occupy the catalytic center of the respective aspartyl intramembrane protease but instead actively participates in the inhibition mechanism by electrostatically blocking the catalytic center of the enzyme is provided by the comparative analysis of NΔE-myc GR and the correspondingly tagged V1744L mutant of NΔE with regard to their ability to block γ-Secretase-dependent processing of NΔE-eGFP (Fig. 5H, I and Suppl. Figure 8 J, K). As demonstrated by others, the V1744L mutant is cleaved much less efficient than wild-type Notch1 and was suggested to be retained longer within the catalytic center of the enzyme complex [40, 52]. Intriguingly, while the V1744L mutant did not inhibit γ-Secretase to a higher degree than wild-type NΔE-myc, the GR variant blocked intramembrane proteolysis even though again being expressed at lower levels. These findings clearly suggest that the inserted PR/RP motif does not simply render NΔE a slow substrate competing for access to the catalytic center with classical substrates, thereby suggesting a similar mechanism in case of Frey1 and SPPL2c.

In principle, transplantation of RP/PR motifs into constitutively cleaved I-CLIP substrates represents a versatile and highly adaptive tool for the generation of protein-based inhibitors of aspartyl intramembrane proteases. Albeit in many cases low molecular weight compounds are available to target these enzymes, most of them lack specificity targeting several family members, though to different extents [17, 53–55]. Intriguingly, generation of inhibitory substrate-based constructs in principle offers striking advantages over classical compound-based inhibition approaches, especially with regard to targeting of single I-CLIPs or even individual protease/substrate pairs. Based on the specific subcellular distribution and membrane topology of the substrate molecule in which the PR/RP motif will be integrated, a more selective targeting is possible since the transplantable motif does not affect the global recognition features of the substrate TMD helix. Based on different affinities of substrates for a shared I-CLIP, even substrate selective approaches are imaginable. However, further experiments will be needed to validate the versatility of the identified targeting approach. One major limitation is represented by the fact that, as evidenced by analysis of Frey1-PLN chimeras as well as NΔE mutants, insertion of the PR/RP motif alone is not sufficient for mediation of inhibition. Instead, this also requires a certain environment within the TMD most likely due to the extensive remodeling of this segment within the catalytic center of the intramembrane protease which is essential for cleavage [31]. In case of NΔE, this motif had to be inserted at a specific position to achieve γ-Secretase inhibition. Additionally, generation of a PLN-based SPPL2c inhibitor required modification of further residues within the transmembrane segment, suggesting that adaptation of this motif for other protease-substrate pairs might require mutational scanning of the transmembrane domain of the selected substrate molecule. Based on the notion that some substrates are cleaved more efficiently than others by intramembrane proteases due to reasons which are only poorly defined in many cases, it might be possible that some substrate TMDs cannot be transformed into inhibitors at all. Nevertheless, the identified motifs and inhibitory mechanisms have the potential to significantly expand our tool box for the analysis of aspartyl I-CLIPs.

An additional interesting extension of our study is that it provides the opportunity to identify yet uncharacterized physiologically relevant inhibitors of aspartic I-CLIPs. To date, Frey1 remains the only characterized protein inhibitor of an aspartic I-CLIP [26]. By contrast, such molecules have been identified for the intramembrane-cleaving metalloprotease SpoIVFB from *Bacillus subtilis* [56], demonstrating that the existence of inhibitory proteins for I-CLIPs might be more common than currently anticipated. The versatility of the identified PR (or the inverted RP) motif demonstrated in our study makes it tempting to speculate that other proteins exist that employ a similar inhibition mode to act as endogenous inhibitors of other aspartic I-CLIPs. In case of SPP/SPPL proteases this would be particularly relevant by providing a possibility for the regulation of the direct cleavage of tail-anchored proteins by these enzymes [20, 47]. Considering presenilins, such specific inhibitors might fulfil potential functions in the suppression of γ-Secretase-related disease conditions including T-ALL and AD. While the identification of Frey1 as inhibitor of SPPL2c was based on the strong downregulation of this protein in the absence of SPPL2 [17] and its inhibition of SPPL2c was not anticipated, our study now provides an objective way to identify potential other protein inhibitors of aspartic I-CLIPs. Beyond the general criteria for substrates of aspartic I-CLIPs including co-localization with the protease, membrane topology and small ectodomains [28], which certainly apply in the case of Frey1, the existence of a membrane-embedded RP/PR sequence (or similar motifs consisting of a positively charged residue and a strong helix-breaker) provides a highly selective criterion for the identification of potential I-CLIP inhibitors, especially due to the generally low abundance of positive charges within TMDs. Future studies will need to address the question if indeed RP/PR motifs in yet non-identified I-CLIP regulators participate in the regulation of the activity of other aspartic I-CLIPs.

In summary, our study provides evidence of the existence of a versatile and transplantable inhibitory motif for aspartic I-CLIPs within the TMD of Frey1, which both significantly expands the tool kit available for studying and modulating these enzymes but also might facilitate the identification of yet unknown physiologically relevant negative regulators of this (patho-)physiologically relevant enzyme family.

## Materials and methods

### Cloning

All primers and constructs employed for this study are listed in the supplementary material and methods section. For all constructs, the murine orfs were utilized if not stated differentially. The constructs encoding HA-tagged RAMP4-2 or HO-1 as well as wild-type SPPL2c-myc or its catalytically inactive mutant SPPL2c D457A-myc have been described previously [17]. Plasmids encoding Notch1ΔE-eGFP and hC99-FLAG were kindly provided by Christoph Kaether (Jena, Germany) or Paul Saftig (Kiel, Germany), respectively. For generation of the wide variety of Frey1 chimeras or mutants, the pcDNA3.1 hygro^(+)^ vector containing the coding sequence of Frey1 (previously called C11orf94 or 1700029I15Rik) equipped with C-terminal 3xFLAG tag generated in Contreras et al. (2022) was used as a backbone. For integration of sequences from either murine PLN or CD74, constructs published in 26 [26] and 13 [13] were utilized as templates. Amplification and modification of open reading frames was carried based on Phusion DNA Polymerase (Thermo Fisher Scientific) based on the protocol suggested by the manufacturer. A 3xFLAG-PLN expressing construct was generated based on the HA-PLN plasmid described by [17]. All coding sequences with their respective tags were ligated into pcDNA 3.1 hygro^(+)^ vector (Thermo Fisher Scientific) using the restriction sites indicated in the primer list and transferred into electrocompetent *Escherichia coli* XL1 Blue cells (Stratagene) by electroporation. Purified plasmids from positive *E. coli* clones were validated by sequencing (Microsynth Seqlab, Göttingen, Germany). Purification of transfection-quality plasmids was performed using the PureYield™ Plasmid Midiprep System (Promega) following the manufacturer’s recommendations.

### Cell culture

HEK293T (DSMZ—German Collection of Microorganisms and Cell Culture GmbH No.: ACC 365) and HeLa (DSMZ No.: ACC 57) cells were maintained in Dulbecco´s modified Eagle´s medium (DMEM + Gluta Max, GIBCO) supplemented with 10% fetal calf serum (Thermo Fisher Scientific), penicillin (100 U/ml) and streptomycin (100 µg/ml, both from Sigma-Aldrich) in an atmosphere of 95% air and 5% CO_2_. For subcultivation or seeding of cells for experiments, monolayers were washed once with phosphate-buffered saline (PBS) and then detached by incubation with Accutase (Thermo Fisher Scientific) for a maximum of 5 min. Cells were finally seeded in the desired concentration depending on the respective application.

### Transfection

HEK293T cells were seeded in six well-plates or 10-cm dishes for total lysis or co-immunoprecipitation experiments, respectively. For immunofluorescence analysis, HeLa cells were grown on coverslips in 12-well plates. On the following day, cells were transfected using polyethyleneimine (PEI MAX Transfection Grade Linear Polyethylenimine, Polysciences), using a DNA:PEI ratio of 1:2.5. In case of transfection with several plasmids at the same time, the total DNA amount was equally divided to the different plasmids. After 6 h, the culture medium was replaced and cells were analyzed on consecutive day.

### Protein extraction and Western blotting

Cells were washed once with PBS before detaching from culture plates with a cell scraper in PBS supplemented with cOmplete protease inhibitor mix (Roche). The cell suspension was centrifuged at 1000×*g* for 5 min; the pellets were resuspended in Lysis Buffer (150 mM NaCl, 50 mM Tris/HCl, 1% (w/v) Triton X-100, 0.1% SDS) in the presence of protease inhibitors [cOmplete protease inhibitor mix, 0.5 µg/ml pepstatin A (Sigma-Aldrich), 4 mM Pefabloc SC Protease Inhibitor (Roth) and 4 mM EDTA], followed by a sonication step. After 1 h on ice, lysates were centrifuged for 10 min at 18,000×*g*. Protein concentration was calculated by Pierce BCA Protein Assay Kit (Thermo Fisher Scientific) following the manufacturer’s recommendations. Samples were prepared for SDS-PAGE with the required amount of 5 × SDS sample buffer [500 mM dithiothreitol, 5% (w/v) SDS, 50% (v/v) glycerol, trace amounts bromophenol blue and 625 mM Tris–HCl (pH 6.8)] and heated 5 min at 56 or 95 °C, when Notch1 constructs were processed. For SDS-PAGE, 10% and 12.5% polyacrylamide gels were used depending on the size of the analyzed proteins. After the electrophoresis of an equal amount of proteins, a semidry transfer to nitrocellulose was performed. Membranes were blocked for 1 h in 5% non-fat milk (Roth) in TBS-T [137 mM NaCl, 2.7 mM KCl, 0.1% (v/v) Tween 20, 25 mM Tris/HCl (pH 7.4)]. After overnight incubation at 4 °C with the respective primary antibody depicted in the individual figures, membranes were washed three times with TBS-T and subsequently incubated for 1 h at RT with the required peroxidase-conjugate secondary antibodies. Dilutions of primary as well as secondary antibodies were prepared in 5% non-fat milk in TBS-T. After three final washes with TBS-T, immunodetection was carried out employing the Amersham ECL Advance Western Blotting Detection Reagent (GE Healthcare) using the Amersham Imager 600 RGB. The following primary antibodies were employed: anti-FLAG M2 (Sigma-Aldrich), anti-SPPL2c (aa672-690, [17]), anti-HA 3F10 (Roche), anti-myc 9B11 (Cell Signaling Technology) or anti-GFP (Cell Signaling Technology) anti-cleaved Notch1 Val1744 D3B8 (Cell Signaling Technology), anti-Cofilin D3F9 (Cell Signaling Technology) and anti-GADPH (BioLegend).

### *γ-Secretase *in vitro* assay*

HEK cells grown in 10-cm dishes were transfected with either NΔE-eGFP or C99-HA together with either NΔE-myc Wt or the corresponding GR mutant. To accumulate the respective substrate molecules, cells were treated for 16 h with 10 µM DAPT. After three washes with cold PBS, cells were scraped of in 1 ml PBS supplemented with cOmplete Protease inhibitor mix and subsequently pelleted for 5 min at 1000 g. The pellet was resuspended in 750 µl hypotonic buffer (10 mM HEPES–NaOH, pH 7.4 + cOmplete protease inhibitor mix) and incubated for 10 min on ice. Afterward, cells were disrupted by passing them eight times through a 27 G cannula. The suspension was spun 5 min at 750*g* at 4 °C, and the post-nuclear supernatants were transferred to an ultracentrifugation tube and membranes were pelleted at 100,000*g* for 1 h at 4 °C. Then, the resulting membrane pellets were resuspended in 150 mM citrate buffer (pH 6.4) containing 1% CHAPSO and cOmplete protease inhibitor mix. Protein concentrations were determined as described above, and samples were adjusted to the same protein concentration. For the actual in vitro assay reaction, 10 µl of the generated membrane solution was mixed with 10 µl reaction mix (for 100: 81.8 µl 150 mM citrate buffer pH 6.4, 2.5 µl cOmplete protease inhibitor mix, 2 µl 1 mM DTT, 3.8 µl 30 mg/ml phosphatidylcholine, 9.9 µl 2 mg/ml BSA). Where indicated, 1 µl 200 µM DAPT were added yielding in a final concentration of 10 µM in the reaction mix. Samples were incubated for 4 h at 37 °C or left on ice as negative control. After this period, the reaction was stopped by supplementing the samples with 5 µl 5 × Laemmli buffer prior to Western Blot analysis of the generated cleavage products.

### Co-immunoprecipitation

To verify the physical interaction of the generated chimeras/mutants and SPPL2c co-immunoprecipitation assays were done. For this purpose, cell lysis was carried out in presence of 0.5% ([3-cholamidopropyl] dimethylammonio)-2-hydroxy-1-propansulfonate (CHAPSO, Roth) and without the sonication step. One milligrams of lysates in a total volume of 500 µl was incubated overnight with 1.0 µl monoclonal anti-FLAG M2 antibody (Sigma) under continuous rotation a 4 °C. After that, 25 µl of equilibrated Protein G agarose beads (Thermo Fisher Scientific) was incubated with the CHAPSO lysates for 2 h under the same conditions. Beads were recovered by centrifugation at 4000×*g* for 1 min and subsequent removal of the supernatant followed by four washing steps with CHAPSO buffer. Elution of the purified immunocomplexes from beads was performed in 2 × SDS sample buffer at 56 °C with shaking for 10 min. Eluates were analyzed by Western Blotting of total lysis versus bead eluate.

### Indirect immunofluorescence

HeLa cells were grown and transfected on coverslips. After 24 h, monolayers were washed three times with PBS and fixed with 4% (w/v) paraformaldehyde (PFA) in PBS for 20 min at RT. After fixation, cells were washed three times with PBS saponine (PBS + 0.2% saponine (Roth)) and then incubated with quenching buffer (0.12% (w/v) glycine in PBS saponine). For blocking, cover slips were incubated for at least 1 h at RT with blocking buffer (1% FCS in PBS saponine). Staining with primary antibodies diluted in blocking buffer was performed overnight at 4 °C in a humid dark chamber. Depending of the protein of interest, the following antibodies were employed: anti-FLAG M2 antibody (Sigma-Aldrich), monoclonal anti-myc 9B11 (Cell Signaling Technology) anti-GM130 clone 35/GM130 (BD Biosciences). After five washing steps with PBS saponine, cover slips were incubated for 1 h at RT with secondary antibodies coupled to either AlexaFluor 488 or AlexaFluor 594 (Molecular Probes) again diluted in blocking buffer. Finally, cover slips were washed five times with PBS saponine and twice with ddH2O and mounted on slides using Mowiol 4-88 (Merck) containing 1,4-diazabicyclo [2.2.2]octane (DABCO, Sigma) and 1 µg/ml 4′,6-diamidino-2-phenylindole (DAPI from Sigma) to visualize nuclei. Pictures were captured using either a Leica SP5 or Leica STELLARIS confocal laser-scanning microscope (Leica) and processed employing ImageJ (version 1.52a) and GNU Image Manipulation Program (version 2.10).

### Structure prediction

Visualization and manipulation of protein structures was performed with the Mol* 3D Viewer (https://www.rcsb.org/3d-view, version 3.23.0) [57] and PyMOL2.5.2 (https://www.pymol.org). Detailed models of the intramembrane proteases and their complexes with substrate TMD chains were generated with the multimer versions of AlphaFold2, accessible through the ColabFold website (https://github.com/sokrypton/ColabFold) [29, 30, 58]. Limited refinement of the final model ensemble was carried out by the Amber program implemented within ColabFold [29].

### Data analysis

Western Blot images were subjected to densitometric analysis employing Image J (version 1.52a). Band intensities were normalized to those of the respective loading control detected from the same membrane. All data were normalized to the respective control samples indicated in the individual figures. The inhibitory capacity of individual Frey1 or PLN mutants was normalized to that of wild-type Frey1. In the case of the analysis of NΔE-eGFP processing in the presence of NΔE-myc inhibitory constructs, band intensities obtained after incubation with anti-cleaved Notch (NICD) were divided by that obtained with a GFP antibody after stripping for the same sample (total reporter protein). Subsequently, NICD/GFP ratios were normalized to that of DMSO-treated control cells. hC99-HA processing from total lysate blots was calculated by densitometric determination of bands intensities for hC99-HA and the additionally accumulating hC83-HA protein. Afterward, band intensities were normalized to that of the employed loading control. All ratios were finally divided by that of the DMSO-treated control samples.

For the quantification of co-immunoprecipitations, band intensities of co-precipitated SPPL2c were divided by those of the corresponding precipitated Frey1 or PLN variant from the same sample. These ratios were then normalized to those obtained for wild-type Frey1.

Where immunofluorescence images were scored, cells were categorized as either presenting dominant nuclear, mixed or dominant plasma membrane localization of the NΔE-eGFP reporter or the overexpressed NΔE-myc variant, respectively. ER staining was not considered for the scoring of the subcellular localization of NΔE-myc variants. Relative amounts of all fractions to the total amount of counted cells were calculated.

All calculations were performed using Excel 2016. Generation of diagrams and statistical analysis were carried out using GraphPad Prism Software (version 8.4.3). The tests employed are depicted in the individual figure legends. All diagrams show mean values ± SD. Statistical significance against the respective control sample is depicted directly above the bar; the significance of other comparisons is indicated by lines between the compared samples. Figures were assembled using Adobe Illustrator CC (version 2015.3.1).

## Supplementary Information

Below is the link to the electronic supplementary material.

Supplementary file1 (DOCX 10306 KB)

Supplementary file1 (PDF 10307 KB)

## Data Availability

All data are available upon request to the corresponding author.
