# Supplementary material for: The transmembrane domain of Frey1 harbors a transplantable inhibitory motif for intramembrane proteases
Source: Cell Mol Life Sci. 2023 Jun 1;80(6):170. doi: 10.1007/s00018-023-04823-7 (PMC10234869; doi:10.1007/s00018-023-04823-7)
Supplement: Supplementary file 1 — Supplementary file1 (DOCX 10306 KB) [file 18_2023_4823_MOESM1_ESM.docx]

**Supplementary Figures**

**
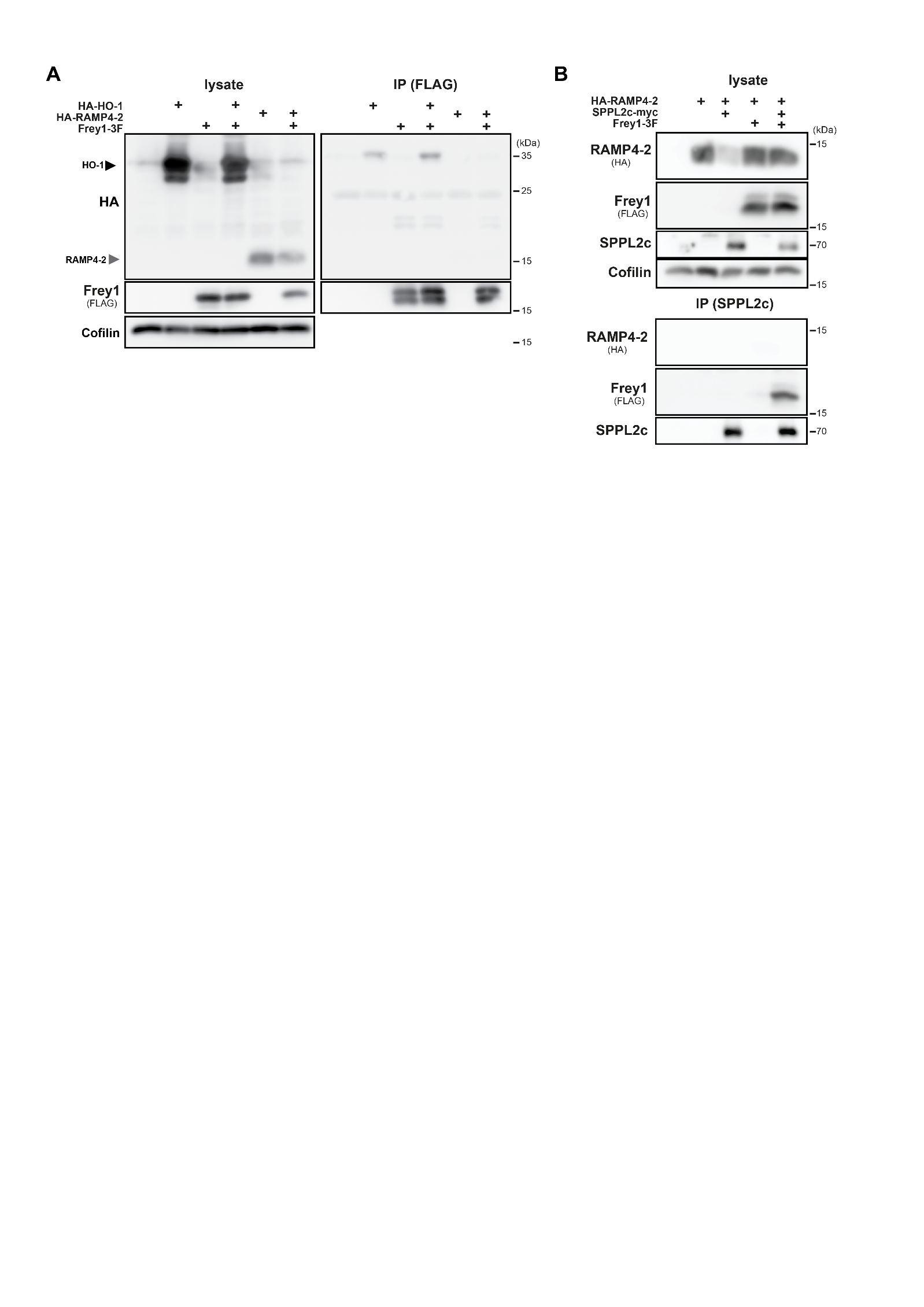
**

**Supplementary Figure 1. Frey1 does not interact with SPPL2c substrates.** **A)** HEK cells were transiently transfected with the HA-tagged SPPL2c substrates HO-1 and RAMP4-2 either alone or together with C-terminally 3xFLAG-tagged Frey1 (Frey1-3F). After cell lysis employing 0.5% CHAPSO, Frey1-3F was precipitated employing anti-FLAG sepharose. Levels of HA-RAMP4-2, HA-HO-1 and Frey-3F both in lysates as well as the immunoprecipitated fraction (IP) were analyzed by Western blotting. The image shows a representative blot from two independent experiments. **B)** SPPL2c-myc was precipitated from CHAPSO lysates of HEK cells transiently transfected with the indicated constructs employing an antibody targeting the C-terminus of the protease. The amounts of HA-RAMP4-2, Frey1-3F and SPPL2c in both lysate and the IP fractions were finally monitored by Western blotting. A single representative experiment from a total of three independent repetitions is depicted.

**
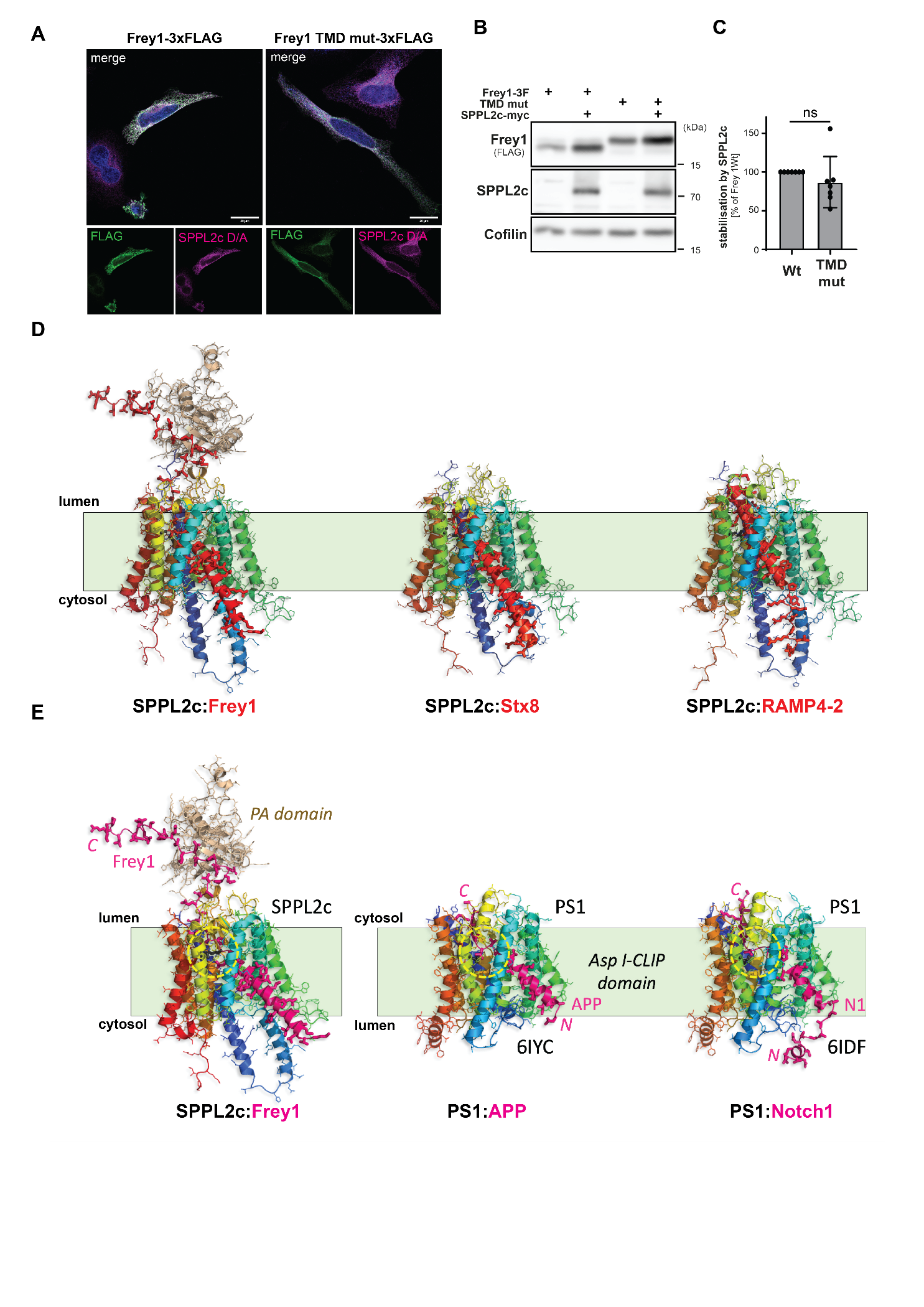
**

**Supplementary Figure 2. Frey1 interacts with SPPL2c in a substrate-like manner.** **A)** The subcellular localization of Frey1-3xFLAG and its TMD mutant (mut) were analyzed by indirect immunofluorescence employing transfected HeLa cells. An inactive variant of SPPL2c-myc (D/A) was co-transfected to validate co-localization of the respective proteins. Scale bar, 20 µm. **B)** Stabilization of Frey1-3xFLAG and the TMD mut variant by SPPL2c-myc was monitored by Western Blotting in transiently transfected HEK cells. **C)** Quantification of E). n=7. Unpaired two-tailed Student’s t-test. ns, not significant. **D)** Using the ColabFold version of AlphaFold2.1-Multimer-multimer (in template-free mode, with Amber relaxation of final model ensemble), the full-length sequence of mouse SPPL2c (UniProt A2A6C4) was paired with Frey1 (UniProt Q8CF31), while for the other peptide substrates, only the transmembrane portion of SPPL2c was used. For STX8 the relevant C-terminal hydrophobic anchor (UniProt O88983, residues 199-236) was extracted, while the 65 residue chain of RAMP4-2 (SERP2, UniProt Q6TAW2) was complexed with SPPL2c. The transmembrane portion of SPPL2c is color-ramped from blue N-term to red C-term, the substrate chains are uniformly colored red. **E)** The full-length sequences of mouse SPPL2c (UniProt identifier A2A6C4) and Frey1 (Q8CF31) were used by AlphaFold2.1-Multimer to build a heterodimeric complex, rendered at left by PyMOL (https://www.pymol.org). The Protease-associated (PA) ectodomain of SPPL2c is colored in wheat, while the TM segment is color-ramped from blue (N-terminus) to red (C-terminus). The bound Frey1 chain is in magenta, with its N-terminal end in the cytosol, and its C-terminal chain wrapped around the PA domain. The yellow dotted circle marks the active site with the two catalytic Asp resides in black, along with the proximal R30 of Frey1. To the right are two PDB structures where the Presenilin-substrate complex has been extracted from the larger γ-Secretase complex for clarity. Presenilin1 bound to either APP (PDB identifier 6IYC) or Notch1 (6IDF) TMDs and have been superposed with SPPL2c, and their active sites are also marked by a yellow dotted line. This aspartyl I-CLIP superposition sets the Presenilin1 chain in the reverse membrane orientation, with the N-terminal end of the substrate chain now in the lumen. The magenta-colored chains of the Presenilin1 substrate TMDs show many residues modeled as alanines that are missing their sidechains because of the moderate resolution of the cryo-EM maps in these areas, while the Frey1 chain is fully modeled.

**
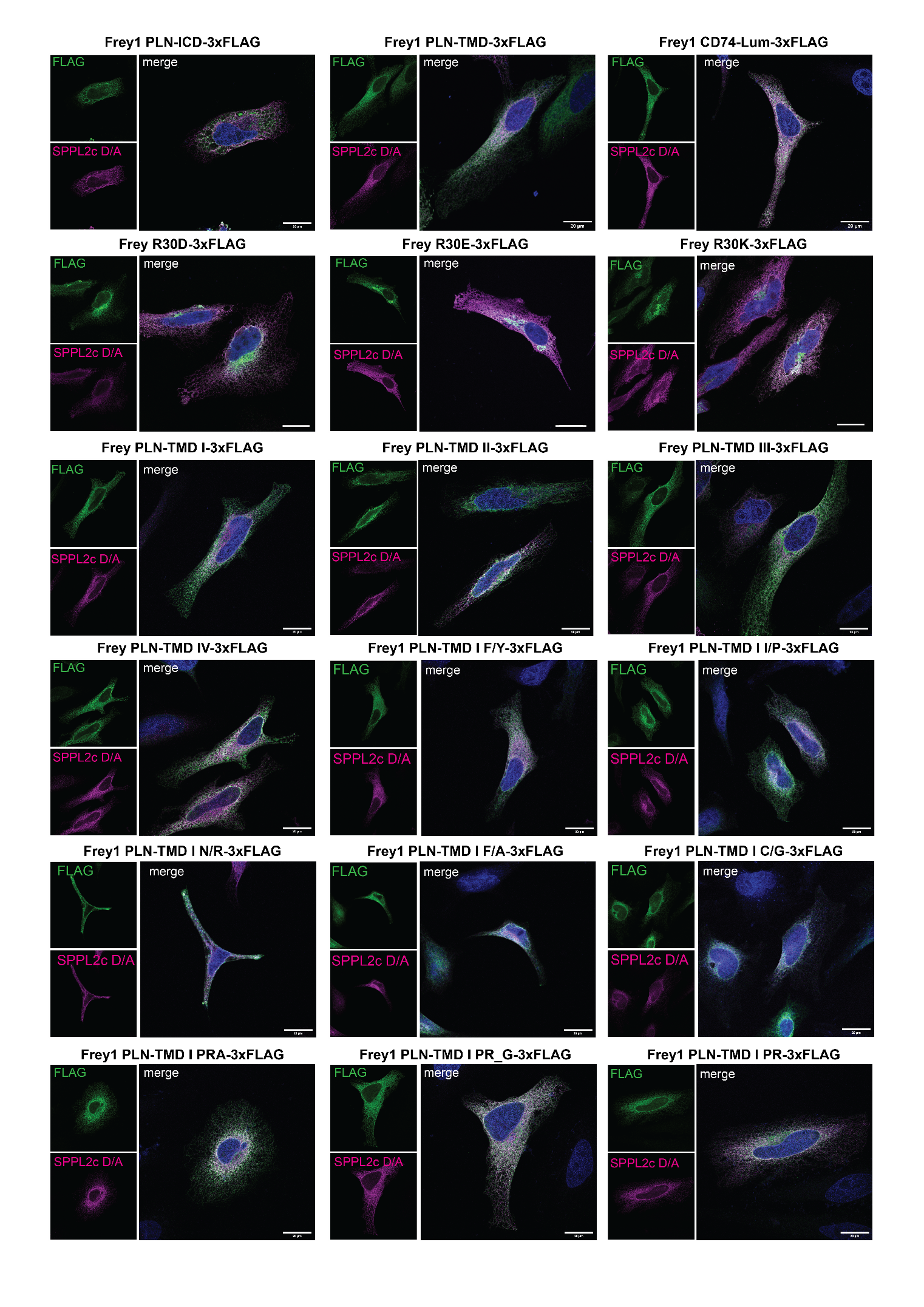
**

**Supplementary Figure 3. Analysis of subcellular localization of the Frey1 mutants generated for this study.** HeLa cells were transiently transfected with the indicated Frey1 variants and an inactive version of SPPL2c (SPPL2c D/A-myc). The subcellular localization of these constructs was finally analyzed by indirect immunofluorescence analysis using FLAG- and myc-targeting antibodies as well as DAPI to stain nuclei. All scale bars, 20 µm.


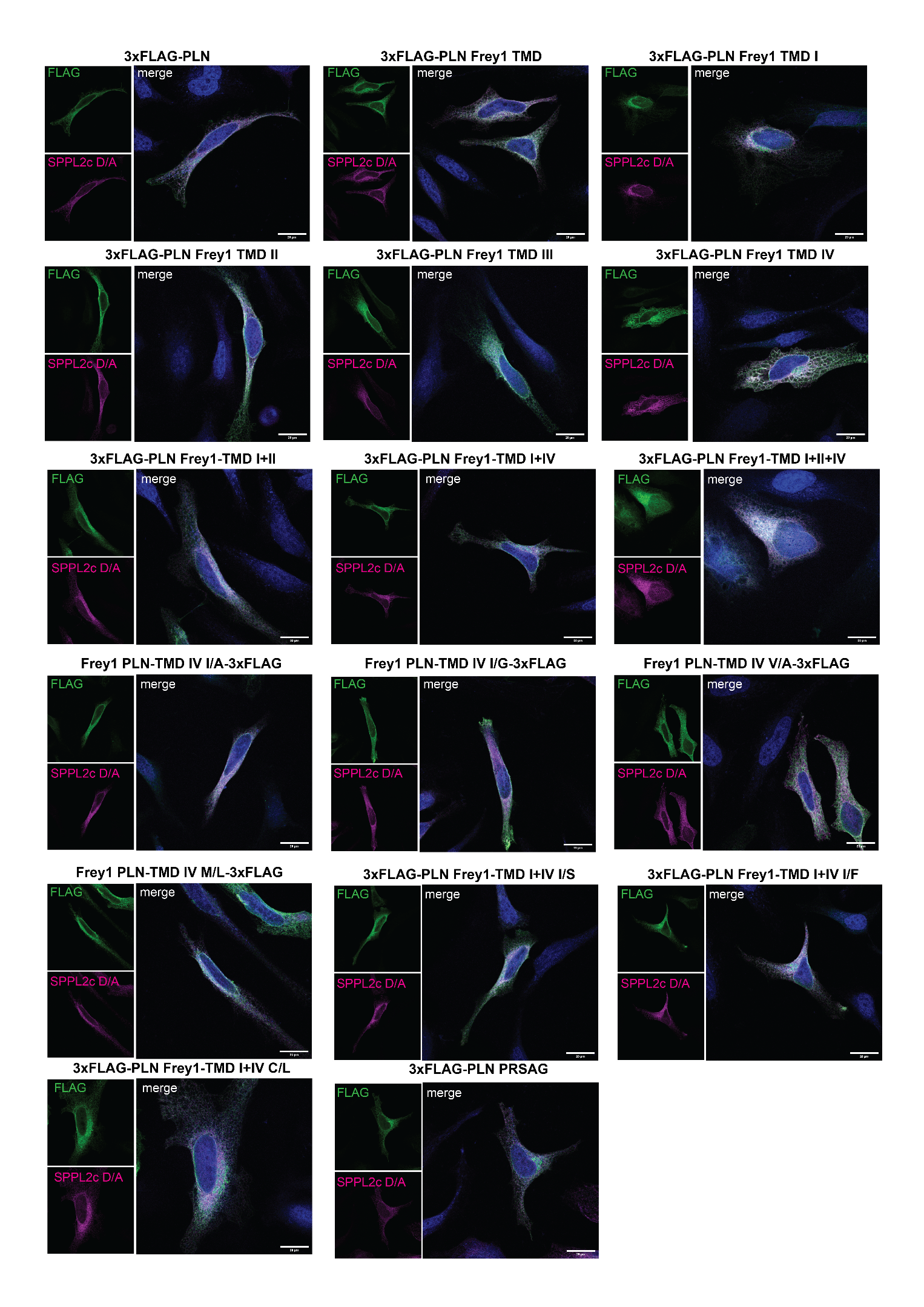


**Supplementary Figure 4. Analysis of subcellular localization of PLN and Frey1-PLN TMD IV mutants generated for this study.** Transiently transfected Hela cells were analyzed for co-localization of 3xFLAG-PLN or Frey1 variants with an inactive mutant of SPPL2c (SPPL2c D/A-myc). Anti-FLAG and anti-myc were employed for staining of PLN variants or SPPL2c, respectively. Nuclei were stained with DAPI. All scale bars, 20 µm.

**
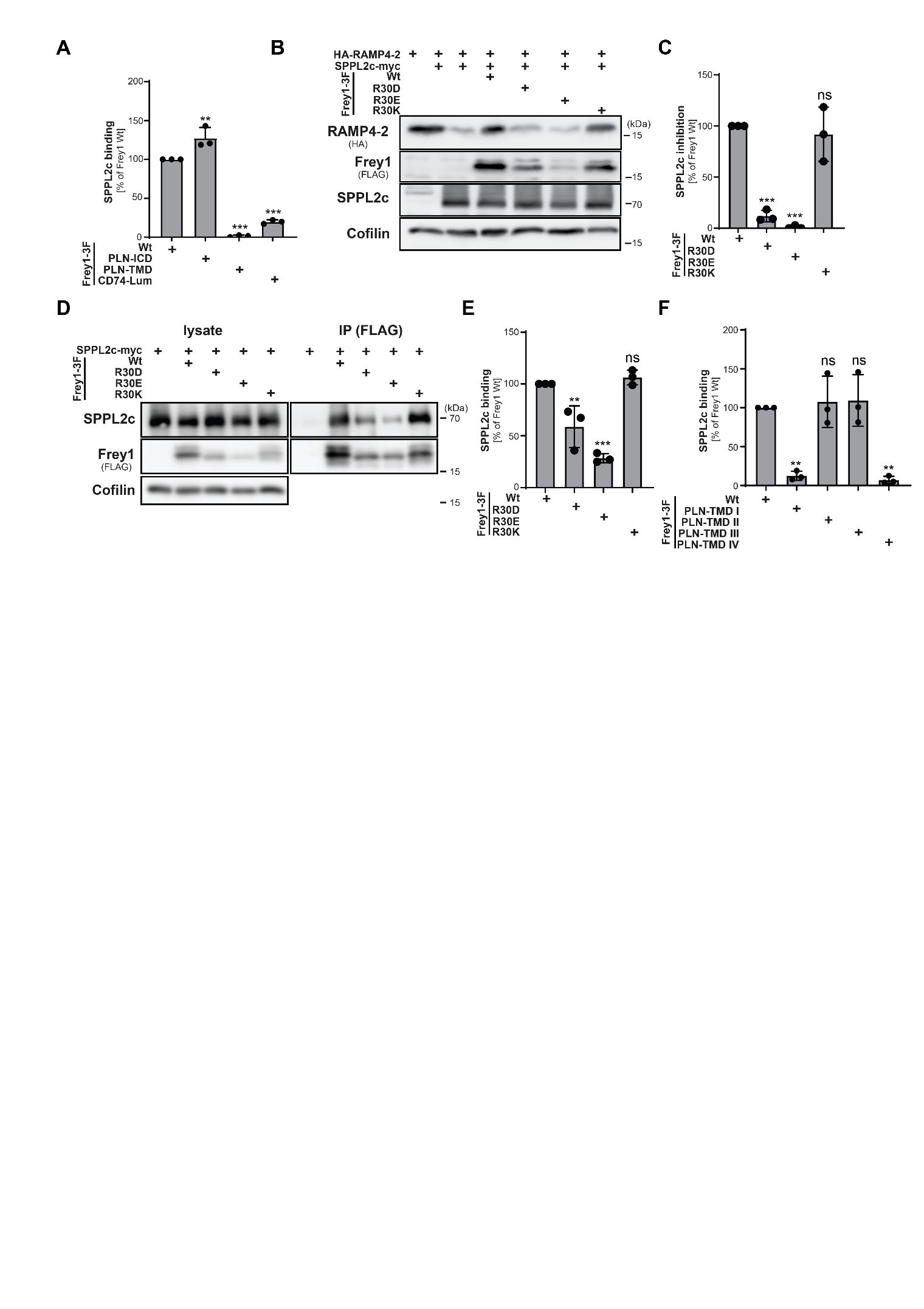
**

**Supplementary Figure 5. The luminal and transmembrane domain of Frey1 contribute to binding and inhibition of SPPL2c. A)** Quantification of binding of Frey1 PLN/CD74 chimeras to SPPL2c-myc as depicted in Fig. 2D. n=3. **B)** HEK cells were transiently transfected with the indicated constructs. Processing of HA-RAMP4-2 by SPPL2c-myc was analyzed by Western Blotting using the indicated antibodies. **C)** Quantification of B). n=3. **D)** Interaction of SPPL2c-myc with Frey1-3xFLAG or its R30D, R30E and R30K mutants was monitored by co-immunoprecipitation experiments employing CHAPSO-lysates of transiently transfected HEK cells. FLAG-tagged proteins were precipitated by corresponding antibodies immobilized on agarose beads. **E)** Quantification of D). n=3. **F)** Quantification of binding of Frey1 PLN-TMD chimeras as depicted in Fig. 2G. n=3. For statistical analysis, in all cases a One-Way ANOVA with Dunnett’s post hoc testing was performed. ns, not significant; ** p≤0.01; *** p≤0.001


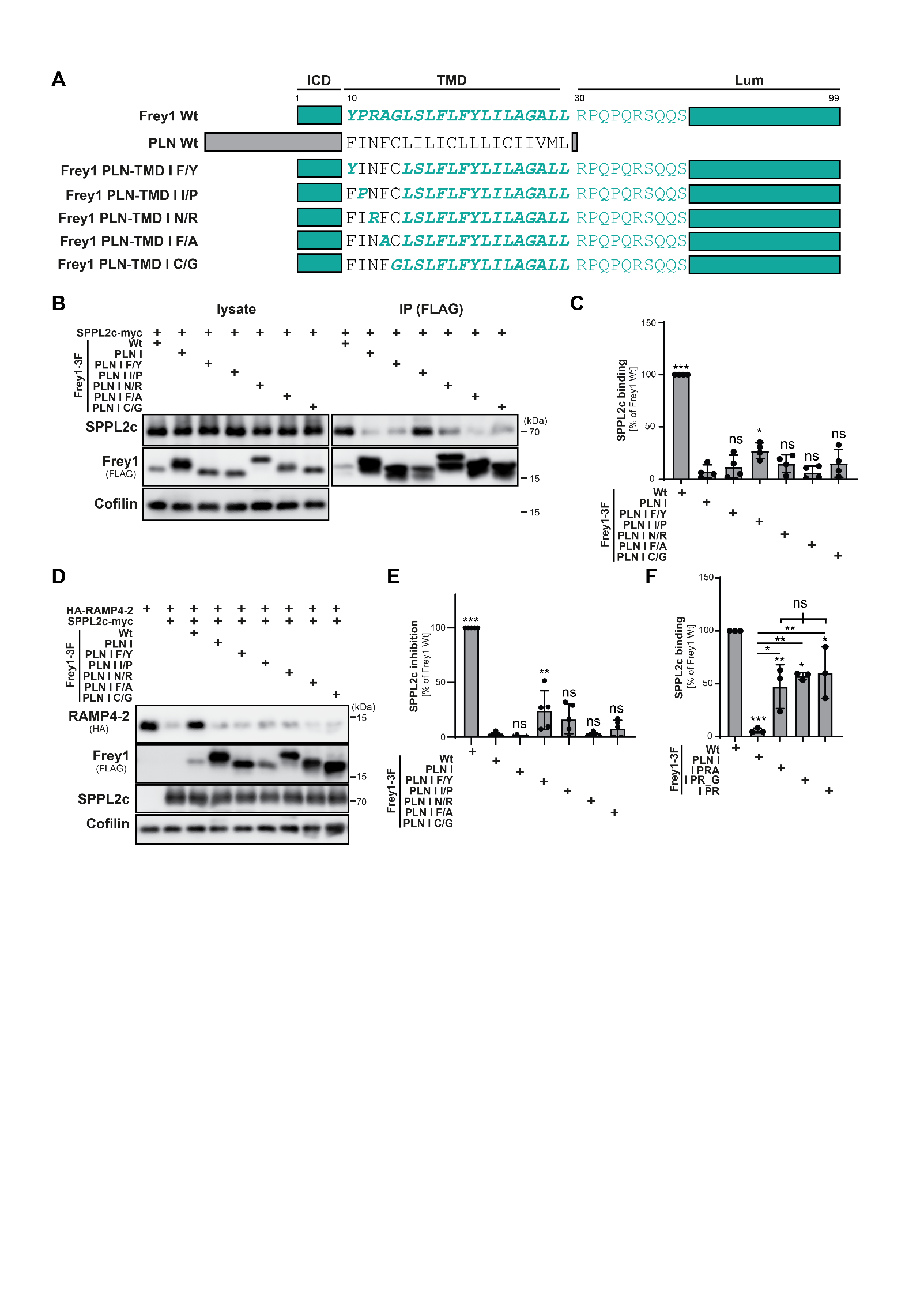


**Supplementary Figure 6. Inhibition of SPPL2c requires a PR motif within the N-terminus of the Frey1 transmembrane domain. A)** Schematic representation of Frey1 mutants specifically utilized for this Figure. **B)** Binding of Frey1 PLN-TMD segment I mutants with re-mutation of individual residues to those of Frey1 to SPPL2c was monitored by Western Blotting following co-immunoprecipitation using FLAG-targeting antibodies. **C)** Quantification of B). n=4. One-way ANOVA followed by Dunnett’s multiple comparisons test. **D)** SPPL2c inhibition assay using the same mutants as in B). **E)** Quantification of D), n=5. One-way ANOVA followed by Dunnett’s multiple comparisons test. **F)** Quantification of SPPL2c-myc levels co-precipitated with the indicated Frey1 variants as depicted in Fig. 2J. n=3. One-Way ANOVA with Tukey’s post hoc testing. ns, not significant; * p≤0.05; ** p≤0.01; *** p≤0.001

**
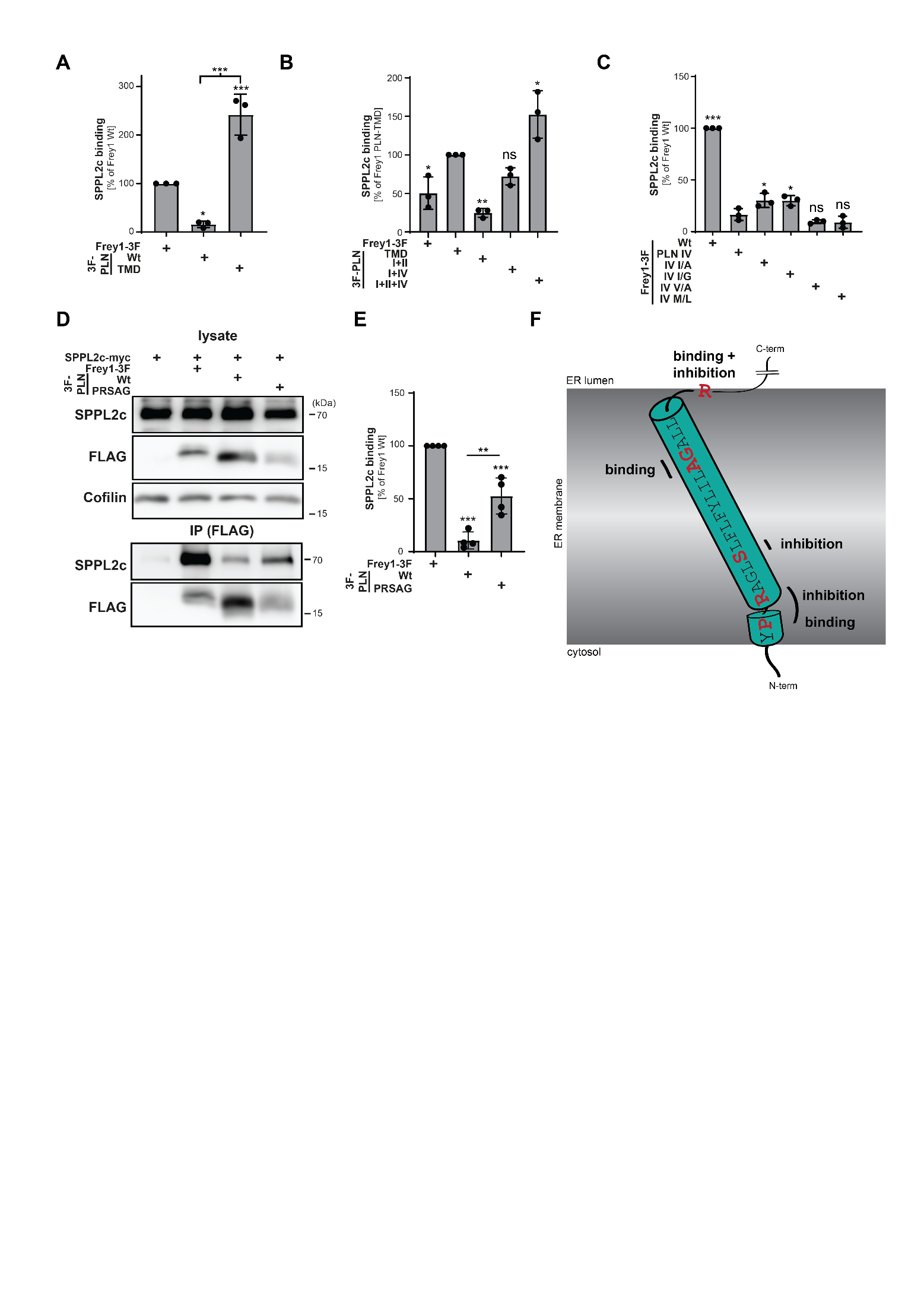
**

**Supplementary Figure 7. Introduction of only five amino acids from the Frey1 TMD are sufficient to transform PLN into a SPPL2c inhibitor. A)** Binding of SPPL2c-myc to PLN/Frey1 chimeras as shown in Fig. 3B was quantified. n=3. One-Way ANOVA with Tukey’s post hoc testing. **B)** Quantification of the co-immunoprecipitation-based binding analysis of SPPL2c-myc with the indicated PLN mutants as depicted in Fig. 4D. n=3. One-Way ANOVA with Dunnett’s multiple comparisons test. **C)** Interaction of SPPL2c-myc with the indicated Frey1-variants as shown in Fig. 4E. n=3. One-Way ANOVA with Dunnett’s multiple comparisons test. **D)** Overview of Frey1 residues involved in SPPL2c inhibition. While the ICD of Frey1 is dispensable for interaction with SPPL2c, both its TMD but also its luminal JMD play central parts in this. R30 within the juxtamembrane domain of Frey1 is required for both interaction and inhibition of SPPL2c most likely due to mediating the initial contact formation of Frey1 and SPPL2c. Within the TMD, especially the N-terminal P11 and R12 residues play essential roles for binding (primarily P11) and inhibition (R12). Additionally, the more C-terminal S16 residue is involved in the mediation of inhibition while the AG motif (residues 25 and 26 of murine Frey1) primarily facilitate the interaction of Frey1 with SPPL2c without substantially affecting the inhibitory potential of Frey1. **E)** HEK cells were transiently transfected with the indicated constructs. After lysis with 0.5% CHAPSO, FLAG-tagged proteins were precipitated employing antibody-conjugated beads. Co-immunoprecipitation of SPPL2c was subsequently analyzed by Western Blotting employing the indicated antibodies. **F)** Quantification of E). n=4. One-Way ANOVA with Tukey’s post hoc testing. ns, not significant; * p≤0.05; ** p≤0.01; *** p≤0.001

**
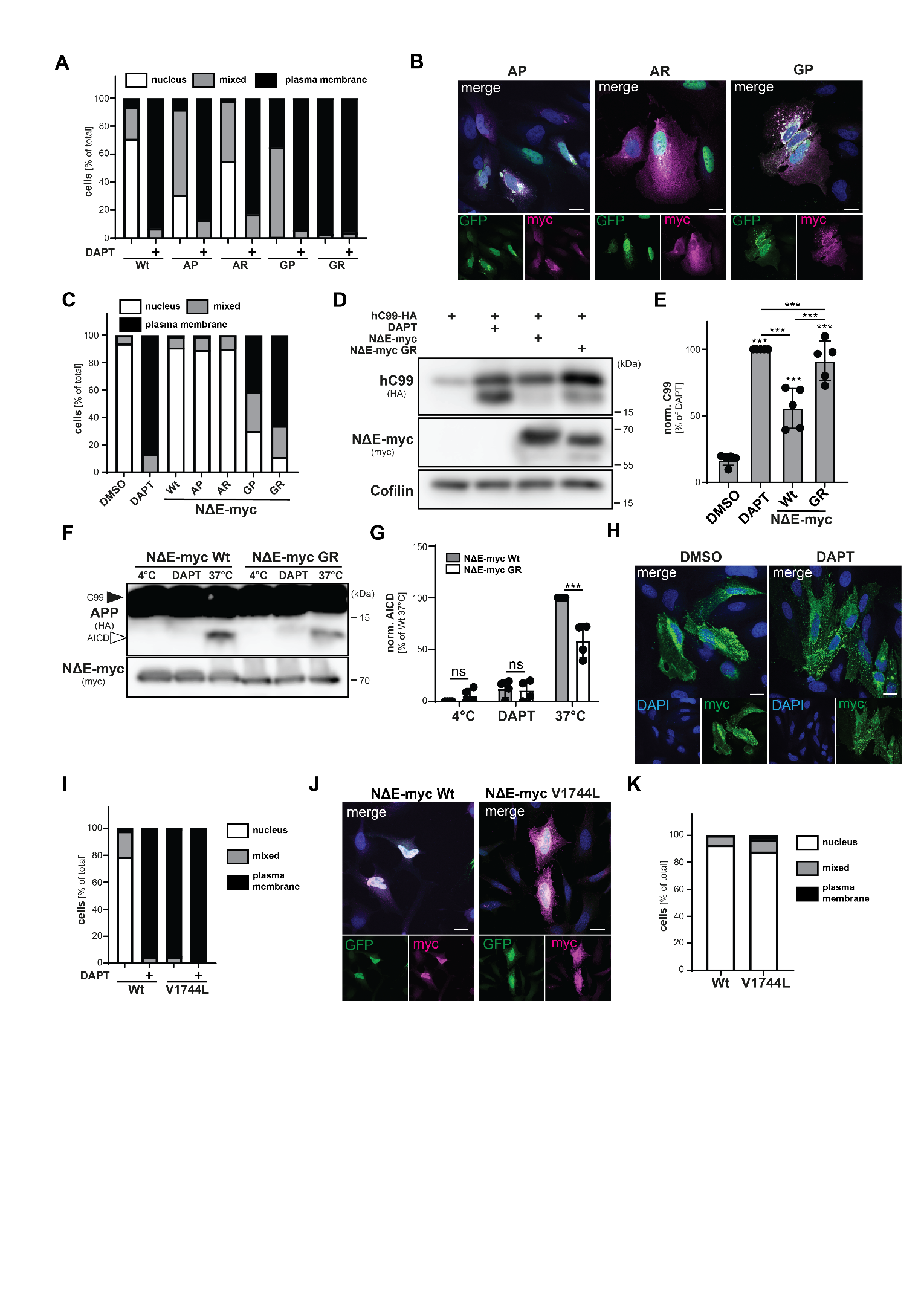
**

**Supplementary Figure 8. The findings from Frey1 and SPPL2c can be transferred to other I-CLIP/substrate pairs. A)** Quantification of the subcellular localization of the indicated NΔE-myc variants as depicted in Fig. 5B. myc-staining was either categorized as predominantly localized within the cell nucleus or at the plasma membrane or as presenting a mixed distribution between these compartments. Per condition, between 65 and 90 cells were evaluated. **B)** HeLa cells were transiently transfected with NΔE-eGFP and the indicated NΔE-myc variants. The subcellular localization of both proteins was finally detected by indirect immunofluorescence. DAPI was employed to visualize nuclei. The presented images supplement those depicted in Fig. 5C. Scale bar, 20 µm. **C)** The immunofluorescence-based γ-Secretase inhibition assays depicted in Fig. 5C and Suppl. Fig. 5B were quantified as described in A). Per condition, between 72 and 85 cells were analyzed for the dominant subcellular localization of the GFP reporter. **D)** HEK cells were transiently transfected with hC99-HA and either wild type (Wt) NΔE-myc or its GR mutant. As control, cells were treated with 10 µM DAPT to block γ-Secretase activity. Accumulation of C99 was subsequently analyzed by Western Blotting. **E)** Quantification of D). n=5. One-Way ANOVA with Tukey’s post hoc testing. **F)** HEK cells overexpressing hC99-HA and the indicated NΔE-myc variants were treated for 16 h with 10 µM DAPT to enrich the γ-Secretase substrate. CHAPSO-solubilized membrane preparations of these cells were subsequently incubated for 6 h either at 4°C as control or at 37°C to allow γ-Secretase-dependent processing of C99. Where indicated, the membrane preparations were incubated in presence of 10 µM DAPT to block enzyme activity. Processing of C99 was finally monitored by Western Blotting. **G)** Quantification of AICD levels as depicted in F). n=4. For statistical analysis, for each condition an unpaired two-tailed Student’s t-test was performed. **H)** HeLa cells were transiently transfected with NΔE-myc V1744L. Where indicated, cells were additionally treated with 10 µM DAPT to block γ-Secretase. After fixation with 4% PFA, the subcellular localization of NΔE-myc V1744L was visualized by indirect immunofluorescence. DAPI was utilized to stain nuclei. Scale bar, 20 µm. **I)** The subcellular localization of NΔE-myc V1744L was quantified as described in A). Per condition, at least 78 cells were analyzed. **J)** The immunofluorescence-based γ-Secretase inhibition assay described in B) was repeated with NΔE-myc Wt or its V1744L mutant. Scale bar, 20 µm. **K)** The data obtained in J) were quantified as described in A). At least 69 cells were analyzed for each condition.
