# Supplementary material for: The transmembrane domain of Frey1 harbors a transplantable inhibitory motif for intramembrane proteases
Source: Cell Mol Life Sci. 2023 Jun 1;80(6):170. doi: 10.1007/s00018-023-04823-7 (PMC10234869; doi:10.1007/s00018-023-04823-7)
Supplement: Supplementary file 2 — Supplementary file1 (PDF 10307 KB) [file 18_2023_4823_MOESM2_ESM.pdf]

construct	template	primers (5` to 3`)	restriction sites
Frey1 PLN-ICD-3xFLAG	pcDNA 3.1 Frey1-3xFLAG	FW: CAAAGCTTATGGAAAAAGTGCAATACCTCACTCGCTCGGCTATCAGGAGAGCCTCCACTATTGAAATGCCTCAGCAAGCACGTCAGAATCTCCAGAACCTATACCCCAGGGCTGGACTC<br>RV: TGACTCGAGTCAAACGGGCCCCTTGTCGTCGTCGTCCTTGTAGTCGATGTCGTGGTCCTTGTAGTCGCCGTCGTGGTCCTTGTAGTCCAGGTTCACATCATAGTAATAGTC	HindIII/XhoI
Frey1 PLN-TMD-3xFLAG	pcDNA 3.1 Frey1-3xFLAG	FW: TCAAAGCTTGCCACCATGGTTCTCGCCATGCTTGGGGCTCTGTTTATCAATTTCTGCCTCATCTTGATATGTCTGCTGCTGATCTGCATCATTGTGATGCTTCGACCTCAGCCCCAGAG<br>RV: TGACTCGAGTCAAACGGGCCCCTTGTCGTCGTCGTCCTTGTAGTCGATGTCGTGGTCCTTGTAGTCGCCGTCGTGGTCCTTGTAGTCCAGGTTCACATCATAGTAATAGTC	HindIII/XhoI
Frey1 CD74 Lum-3xFLAG	pcDNA 3.1 HA-CD74	FW: TCAGGATCCATGGTTCTCGCCATGCTTGGGGCTCTGTACCCCAGGGCTGGACTCAGTCTCTTCCTTTTTTACCTCATCTTGGCAGGCGCACTCCTTCAGCAACAGGGCCGCCTAGAC<br>RV: TGACTCGAGTCAAACGGGCCCCTTGTCGTCGTCGTCCTTGTAGTCGATGTCGTGGTCCTTGTAGTCGCCGTCGTGGTCCTTGTAGTCCACATGGTCCTGGGTCATGTTGCC	BamHI/XhoI
Frey1 R30D-3xFLAG	pcDNA 3.1 Frey1-3xFLAG	FW: TCAAAGCTTATGGTTCTCGCCATGCTTGGGGCTCTGTACCCCAGGGCTGGACTCAGTCTCTTCCTTTTTTACCTCATCTTGGCAGGCGCACTCCTTGATCCTCAGCCCCAGAGGTCTC<br>RV: TGACTCGAGTCAAACGGGCCCCTTGTCGTCGTCGTCCTTGTAGTCGATGTCG	HindIII/XhoI
Frey1 R30E-3xFLAG	pcDNA 3.1 Frey1-3xFLAG	FW: TCAAAGCTTATGGTTCTCGCCATGCTTGGGGCTCTGTACCCCAGGGCTGGACTCAGTCTCTTCCTTTTTTACCTCATCTTGGCAGGCGCACTCCTTGAGCCTCAGCCCCAGAGGTCTC<br>RV: TGACTCGAGTCAAACGGGCCCCTTGTCGTCGTCGTCCTTGTAGTCGATGTCG	HindIII/XhoI
Frey1 R30K-3xFLAG	pcDNA 3.1 Frey1-3xFLAG	FW: TCAAAGCTTATGGTTCTCGCCATGCTTGGGGCTCTGTACCCCAGGGCTGGACTCAGTCTCTTCCTTTTTTACCTCATCTTGGCAGGCGCACTCCTTAAACCTCAGCCCCAGAGGTCTC<br>RV: TGACTCGAGTCAAACGGGCCCCTTGTCGTCGTCGTCCTTGTAGTCGATGTCG	HindIII/XhoI
Frey1 PLN-TMD I-3xFLAG	pcDNA 3.1 Frey1-3xFLAG	FW: TCAAAGCTTGCCACCATGGTTCTCGCCATGCTTGGGGCTCTGTTTATCAATTTCTGCCTCAGTCTCTTCCTTTTTTACC<br>RV: TGACTCGAGTCAAACGGGCCCCTTGTCGTCGTCGTCCTTGTAGTCGATGTCGTGGTCCTTGTAGTCGCCGTCGTGGTCCTTGTAGTCCAGGTTCACATCATAGTAATAGTC	HindIII/XhoI
Frey1 PLN-TMD II-3xFLAG	pcDNA 3.1 Frey1-3xFLAG	FW: TCAAAGCTTGCCACCATGGTTCTCGCCATGCTTGGGGCTCTGTACCCCAGGGCTGGACTCATCTTGATATGTTTTTACCTCATCTTGGC<br>RV: TGACTCGAGTCAAACGGGCCCCTTGTCGTCGTCGTCCTTGTAGTCGATGTCGTGGTCCTTGTAGTCGCCGTCGTGGTCCTTGTAGTCCAGGTTCACATCATAGTAATAGTC	HindIII/XhoI
Frey1 PLN-TMD III-3xFLAG	pcDNA 3.1 Frey1-3xFLAG	FW: TCAAAGCTTGCCACCATGGTTCTCGCCATGCTTGGGGCTCTGTACCCCAGGGCTGGACTCAGTCTCTTCCTTCTGCTGCTGATCTGCGCAGGCGCACTCCTTC<br>RV: TGACTCGAGTCAAACGGGCCCCTTGTCGTCGTCGTCCTTGTAGTCGATGTCGTGGTCCTTGTAGTCGCCGTCGTGGTCCTTGTAGTCCAGGTTCACATCATAGTAATAGTC	HindIII/XhoI
Frey1 PLN-TMD IV-3xFLAG	pcDNA 3.1 Frey1-3xFLAG	FW: TCAAAGCTTGCCACCATGGTTCTCGCCATGCTTGGGGCTCTGTACCCCAGGGCTGGACTCAGTCTCTTCCTTTTTTACCTCATCTTGATCATTGTGATGCTTCGACCTCAGCCCCAGAG<br>RV: TGACTCGAGTCAAACGGGCCCCTTGTCGTCGTCGTCCTTGTAGTCGATGTCGTGGTCCTTGTAGTCGCCGTCGTGGTCCTTGTAGTCCAGGTTCACATCATAGTAATAGTC	HindIII/XhoI
Frey1 PLN-TMD I F/Y-3xFLAG	pcDNA 3.1 Frey1 PLN-TMD I-3xFLAG	FW: TCAAAGCTTGCCACCATGGTTCTCGCCATGCTTGGGGCTCTGTATATCAATTTCTGCCTCAGTC<br>RV: TGACTCGAGTCAAACGGGCCCCTTGTCGTCGTCGTCCTTGTAGTCGATGTCGTGGTCCTTGTAGTCGCCGTCGTGGTCCTTGTAGTCCAGGTTCACATCATAGTAATAGTC	HindIII/XhoI
Frey1 PLN-TMD I I/P-3xFLAG	pcDNA 3.1 Frey1 PLN-TMD I-3xFLAG	FW: TCAAAGCTTGCCACCATGGTTCTCGCCATGCTTGGGGCTCTGTTTCCCAATTTCTGCCTCAGTCTC<br>RV: TGACTCGAGTCAAACGGGCCCCTTGTCGTCGTCGTCCTTGTAGTCGATGTCGTGGTCCTTGTAGTCGCCGTCGTGGTCCTTGTAGTCCAGGTTCACATCATAGTAATAGTC	HindIII/XhoI
Frey1 PLN-TMD I N/R-3xFLAG	pcDNA 3.1 Frey1 PLN-TMD I-3xFLAG	FW: TCAAAGCTTGCCACCATGGTTCTCGCCATGCTTGGGGCTCTGTTTATCAGGTTCTGCCTCAGTCTCTTC<br>RV:TGACTCGAGTCAAACGGGCCCCTTGTCGTCGTCGTCCTTGTAGTCGATGTCGTGGTCCTTGTAGTCGCCGTCGTGGTCCTTGTAGTCCAGGTTCACATCATAGTAATAGTC	HindIII/XhoI
Frey1 PLN-TMD I F/A-3xFLAG	pcDNA 3.1 Frey1 PLN-TMD I-3xFLAG	FW: TCAAAGCTTGCCACCATGGTTCTCGCCATGCTTGGGGCTCTGTTTATCAATGCCTGCCTCAGTCTCTTCCTTTTTTAC<br>RV: TGACTCGAGTCAAACGGGCCCCTTGTCGTCGTCGTCCTTGTAGTCGATGTCGTGGTCCTTGTAGTCGCCGTCGTGGTCCTTGTAGTCCAGGTTCACATCATAGTAATAGTC	HindIII/XhoI
Frey1 PLN-TMD I C/G-3xFLAG	pcDNA 3.1 Frey1 PLN-TMD I-3xFLAG	FW: TCAAAGCTTGCCACCATGGTTCTCGCCATGCTTGGGGCTCTGTTTATCAATTTCGGCCTCAGTCTCTTCCTTTTTTACC<br>RV: TGACTCGAGTCAAACGGGCCCCTTGTCGTCGTCGTCCTTGTAGTCGATGTCGTGGTCCTTGTAGTCGCCGTCGTGGTCCTTGTAGTCCAGGTTCACATCATAGTAATAGTC	HindIII/XhoI
Frey1 PLN-TMD I PRA-3xFLAG	pcDNA 3.1 Frey1 PLN-TMD I-3xFLAG	FW: TCAAAGCTTGCCACCATGGTTCTCGCCATGCTTGGGGCTCTGTTTCCCAGGGCCTGCCTCAGTCTCTTCC<br>RV: TGACTCGAGTCAAACGGGCCCCTTGTCGTCGTCGTCCTTGTAGTCGATGTCGTGGTCCTTGTAGTCGCCGTCGTGGTCCTTGTAGTCCAGGTTCACATCATAGTAATAGTC	HindIII/XhoI
Frey1 PLN-TMD I PRxG-3xFLAG	pcDNA 3.1 Frey1 PLN-TMD I-3xFLAG	FW: TCAAAGCTTGCCACCATGGTTCTCGCCATGCTTGGGGCTCTGTTTCCCAGGTTCGGCCTCAGTCTCTTCCTTTTTTACC<br>RV: TGACTCGAGTCAAACGGGCCCCTTGTCGTCGTCGTCCTTGTAGTCGATGTCGTGGTCCTTGTAGTCGCCGTCGTGGTCCTTGTAGTCCAGGTTCACATCATAGTAATAGTC	HindIII/XhoI
Frey1 PLN-TMD I PR-3xFLAG	pcDNA 3.1 Frey1 PLN-TMD I-3xFLAG	FW: TCAAAGCTTGCCACCATGGTTCTCGCCATGCTTGGGGCTCTGTTTCCCAGGTTCTGCCTCAGTCTCTTCC<br>RV: TGACTCGAGTCAAACGGGCCCCTTGTCGTCGTCGTCCTTGTAGTCGATGTCGTGGTCCTTGTAGTCGCCGTCGTGGTCCTTGTAGTCCAGGTTCACATCATAGTAATAGTC	HindIII/XhoI
Frey1 PLN-TMD IV I/A-3xFLAG	pcDNA 3.1 Frey1 PLN-TMD IV-3xFLAG	FW: TCAAAGCTTGCCACCATGGTTCTCGCCATGCTTGGGGCTCTGTACCCCAGGGCTGGACTCAGTCTCTTCCTTTTTTACCTCATCTTGGCAATTGTGATGCTTCGACCTCAGC<br>RV: TGACTCGAGTCAAACGGGCCCCTTGTCGTCGTCGTCCTTGTAGTCGATGTCGTGGTCCTTGTAGTCGCCGTCGTGGTCCTTGTAGTCCAGGTTCACATCATAGTAATAGTC	HindIII/XhoI
Frey1 PLN-TMD IV I/G-3xFLAG	pcDNA 3.1 Frey1 PLN-TMD IV-3xFLAG	FW: CAAAGCTTGCCACCATGGTTCTCGCCATGCTTGGGGCTCTGTACCCCAGGGCTGGACTCAGTCTCTTCCTTTTTTACCTCATCTTGATCGGCGTGATGCTTCGACCTCAGCC<br>RV: TGACTCGAGTCAAACGGGCCCCTTGTCGTCGTCGTCCTTGTAGTCGATGTCGTGGTCCTTGTAGTCGCCGTCGTGGTCCTTGTAGTCCAGGTTCACATCATAGTAATAGTC	HindIII/XhoI
Frey1 PLN-TMD IV V/A-3xFLAG	pcDNA 3.1 Frey1 PLN-TMD IV-3xFLAG	FW: TCAAAGCTTGCCACCATGGTTCTCGCCATGCTTGGGGCTCTGTACCCCAGGGCTGGACTCAGTCTCTTCCTTTTTTACCTCATCTTGATCATTGCAATGCTTCGACCTCAGCCCCAG<br>RV: TGACTCGAGTCAAACGGGCCCCTTGTCGTCGTCGTCCTTGTAGTCGATGTCGTGGTCCTTGTAGTCGCCGTCGTGGTCCTTGTAGTCCAGGTTCACATCATAGTAATAGTC	HindIII/XhoI
Frey1 PLN TMD IV M/L-3xFLAG	pcDNA 3.1 Frey1 PLN-TMD IV-3xFLAG	FW: TCAAAGCTTGCCACCATGGTTCTCGCCATGCTTGGGGCTCTGTACCCCAGGGCTGGACTCAGTCTCTTCCTTTTTTACCTCATCTTGATCATTGTGCTCCTTCGACCTCAGCCCCAG<br>RV: TGACTCGAGTCAAACGGGCCCCTTGTCGTCGTCGTCCTTGTAGTCGATGTCGTGGTCCTTGTAGTCGCCGTCGTGGTCCTTGTAGTCCAGGTTCACATCATAGTAATAGTC	HindIII/XhoI
3xFLAG-PLN	pcDNA 3.1 HA-PLN	FW: GCAAGCTTGCCACCATGGACTACAAGGACCACGACGGCGACTACAAGGACCACGACATCGACTACAAGGACGACGACGACAAGGGGCCCGTTGAAAAAGTGCAATACCTC<br>RV: GCATCTCGAGTCACAGAAGCATCACAATGATGC	HindIII/XhoI
3xFLAG-PLN Frey1-TMD	pcDNA 3.1 HA-PLN	FW: GCAAGCTTGCCACCATGGACTACAAGGACCACGACGGCGACTACAAGGACCACGACATCGACTACAAGGACGACGACGACAAGGGGCCCGTTGAAAAAGTGCAATACCTC<br>RV: GCATCTCGAGTCACAGAAGGAGTGCGCCTGCCAAGATGAGGTAAAAAAGGAAGAGACTGAGTCCAGCCCTGGGGTA	HindIII/XhoI
3xFLAG-PLN Frey1-TMD I	pcDNA 3.1 3xFLAG-PLN	FW: GCAAGCTTGCCACCATGGACTACAAGGACCACGACGGCGACTACAAGGACCACGACATCGACTACAAGGACGACGACGACAAGGGGCCCGTTGAAAAAGTGCAATACC<br>RV:GCATCTCGAGTCACAGAAGCATCACAATGATGCAGATCAGCAGCAGACATATCAAGATGAGTCCAGCCCTGGGGTATAGGTTCTGGAGAT	HindIII/XhoI
3xFLAG-PLN Frey1-TMD II	pcDNA 3.1 3xFLAG-PLN	FW: GCAAGCTTGCCACCATGGACTACAAGGACCACGACGGCGACTACAAGGACCACGACATCGACTACAAGGACGACGACGACAAGGGGCCCGTTGAAAAAGTGCAATACC<br>RV: GCATCTCGAGTCACAGAAGCATCACAATGATGCAGATCAGCAGCAGAAGGAAGAGACTGAGGCAGAAATTGATAAATAGG	HindIII/XhoI
3xFLAG-PLN Frey1-TMD III	pcDNA 3.1 3xFLAG-PLN	FW: GCAAGCTTGCCACCATGGACTACAAGGACCACGACGGCGACTACAAGGACCACGACATCGACTACAAGGACGACGACGACAAGGGGCCCGTTGAAAAAGTGCAATACC<br>RV: GCATCTCGAGTCACAGAAGCATCACAATGATCAAGATGAGGTAAAAACATATCAAGATGAGGC	HindIII/XhoI
3xFLAG-PLN Frey1-TMD IV	pcDNA 3.1 3xFLAG-PLN	FW: GCAAGCTTGCCACCATGGACTACAAGGACCACGACGGCGACTACAAGGACCACGACATCGACTACAAGGACGACGACGACAAGGGGCCCGTTGAAAAAGTGCAATACC<br>RV: GCATCTCGAGTCACAGAAGGAGTGCGCCTGCGCAGATCAGCAGCAGAC	HindIII/XhoI
3xFLAG-PLN Frey1-TMD I+IV	pcDNA 3.1 3xFLAG-PLN Frey1-TMD I	FW: GCAAGCTTGCCACCATGGACTACAAGGACCACGACGGCGACTACAAGGACCACGACATCGACTACAAGGACGACGACGACAAGGGGCCCGTTGAAAAAGTGCAATACC<br>Rv: GCATCTCGAGTCACAGAAGGAGTGCGCCTGCGCAGATCAGCAGCAGAC	HindIII/XhoI
3xFLAG-PLN Frey1-TMD I+II	pcDNA 3.1 3xFLAG-PLN Frey1-TMD I	Fw: GCAAGCTTGCCACCATGGACTACAAGGACCACGACGGCGACTACAAG<br>Rv: GCATCTCGAGTCACAGAAGCATCACAATGATGCAGATCAGCAGCAGAAGGAAGAGACTGAGTCCAGCCCTGGGGTATAGGTTCTGGAGATTCTGACG	HindIII/XhoI
3xFLAG-PLN Frey1-TMD I+II+IV	pcDNA 3.1 3xFLAG PLN-Frey1 TMD I+IV	Fw: GCAAGCTTGCCACCATGGACTACAAGGACCACGACGGCGACTAC<br>Rv: GCATCTCGAGTCACAGAAGGAGTGCGCCTGCGCAGATCAGCAGCAGAAGGAAGAGACTGAGTCCAG	HindIII/XhoI
3xFLAG PLN-Frey1 TMD I+IV I/S	pcDNA 3.1 3xFLAG PLN-Frey1 TMD I+IV	GCAAGCTTGCCACCATGGACTACAAGGACCACGACGGCGACTACAAG<br>GCATCTCGAGTCACAGAAGGAGTGCGCCTGCGCAGATCAGCAGCAGACATATCAAACTGAGTCCAGCCCTGGGGTATAG	HindIII/XhoI
3xFLAG PLN-Frey1 TMDI+IV I/F	pcDNA 3.1 3xFLAG PLN-Frey1 TMD I+IV	GCAAGCTTGCCACCATGGACTACAAGGACCACGACGGCGACTACAAG<br>GCATCTCGAGTCACAGAAGGAGTGCGCCTGCGCAGATCAGCAGCAGACAGAACAAGATGAGTCCAGCCCTG	HindIII/XhoI
3xFLAG PLN-Frey1 TMD I+IV C/L	pcDNA 3.1 3xFLAG PLN-Frey1 TMD I+IV	GCAAGCTTGCCACCATGGACTACAAGGACCACGACGGCGACTACAAG<br>GCATCTCGAGTCACAGAAGGAGTGCGCCTGCGCAGATCAGCAGCAGAAGTATCAAGATGAGTCCAGCC	HindIII/XhoI
3xFLAG-PLN Frey1-TMD PRSAG	pcDNA 3.1 3xFLAG-PLN PR	GCAAGCTTGCCACCATGGACTACAAGGACCACGACGGCGACTACAAG<br>GCATCTCGAGTCACAGAAGCATCACGCCTGCGCAGATCAGCAGCAGACATATCAAACTGAGGCAGAACCTGGGAAATAG	HindIII/XhoI
NotchΔE-WT-myc	pcDNA3.1 NotchΔE -eGFP	FW: ATCAAGCTTGCCACCATGCCACGGCTCCTGACGCCCTTG<br>RV: GGATCCTTACAGATCCTCTTCTGAGATGAGTTTTTGTTCGTCTAGACTCGAGCTGTCCAAC	HindIII/BamHI
NotchΔE-AP-myc	pcDNA3.1 NotchΔE-WT-myc	FW1: ATCAAGCTTGCCACCATGCCACGGCTCCTGACGCCCTTG<br>RV1: CCACAAAGAACAGGAGCACGAAGGCGCGCGGTGCCACGTACACGAGGTGC<br>FW2: GCACCTCGTGTACGTGGCACCGCGCGCCTTCGTGCTCCTGTTCTTTGTGG<br>RV2: GGATCCTTACAGATCCTCTTCTGAGATGAGTTTTTGTTCGTCTAGACTCGAGCTGTCCAAC	HindIII/BamHI
NotchΔE-AR-myc	pcDNA3.1 NotchΔE-WT-myc	FW1: ATCAAGCTTGCCACCATGCCACGGCTCCTGACGCCCTTG<br>RV1: CCACAAAGAACAGGAGCACGAAGGCCGGGCGTGCCACGTACACGAGGTGC<br>FW2: GCACCTCGTGTACGTGGCACGCCCGGCCTTCGTGCTCCTGTTCTTTGTGG<br>RV2: GGATCCTTACAGATCCTCTTCTGAGATGAGTTTTTGTTCGTCTAGACTCGAGCTGTCCAAC	HindIII/BamHI
NotchΔE-GP-myc	pcDNA3.1 NotchΔE-WT-myc	FW1: ATCAAGCTTGCCACCATGCCACGGCTCCTGACGCCCTTG<br>RV1: CCTCGCGCTTGCGGGACAGCAGCCGCGGACAGCCCACAAAGAAC<br>FW2: GTTCTTTGTGGGCTGTCCGCGGCTGCTGTCCCGCAAGCGCGAGG<br>RV2: GGATCCTTACAGATCCTCTTCTGAGATGAGTTTTTGTTCGTCTAGACTCGAGCTGTCCAAC	HindIII/BamHI
NotchΔE-GR-myc	pcDNA3.1 NotchΔE-WT-myc	FW1: ATCAAGCTTGCCACCATGCCACGGCTCCTGACGCCCTTG<br>RV1: CCTCGCGCTTGCGGGACAGCAGCGGGCGACAGCCCACAAAGAAC<br>FW2: GTTCTTTGTGGGCTGTCGCCCGCTGCTGTCCCGCAAGCGCGAGG<br>RV2: GGATCCTTACAGATCCTCTTCTGAGATGAGTTTTTGTTCGTCTAGACTCGAGCTGTCCAAC	HindIII/BamHI
hC99-HA	pcDNA3.1 hC99-FLAG	FW: ACGAAGCTTGCCACCATGGATGCAGAATTCCGACATGAC<br>RV: GCTCTCGAGCTAAGCGTAGTCTGGGACGTCGTATGGGTAGTTCTGCATCTGCTCAAAG	HindIII/XhoI
NotchΔE-V1744L-myc	pcDNA3.1 NotchΔE-WT-myc	FW1: ATCAAGCTTGCCACCATGCCACGGCTCCTGACGC<br>RV1: GATGGATCCTTACAGATCCTCTTCTGAGATGAGTTTTTGTTCGTC<br>FW2: CTGTTCTTTGTGGGCTGTGGGCTGCTGCTGTCCCGCAAGCGCGAG<br>RV2: CTCGCGCTTGCGGGACAGCAGCAGCCCACAGCCCACAAAGAACAG	HindIII/BamHI

Published constructs

construct	vector	publication	
HA-HO1	pcDNA3.1 hygro(+)	Niemeyer et al., 2019	
HA-RAMP4-2	pcDNA3.1 hygro(+)	Niemeyer et al., 2019	
mSPPL2c-myc	pcDNA3.1 hygro(+)	Niemeyer et al., 2019	
mSPPL2c-myc D457A	pcDNA3.1 hygro(+)	Niemeyer et al., 2019	
HA-PLN	pcDNA3.1 hygro(+)	Niemeyer et al., 2019	
HA-CD74	pcDNA3.1 hygro(+)	Schneppenheim et al., 2013	
